# Gender stereotypes embedded in natural language are stronger in more economically developed and individualistic countries

**DOI:** 10.1093/pnasnexus/pgad355

**Published:** 2023-11-21

**Authors:** Clotilde Napp

**Affiliations:** CNRS, UMR7088, France; Université Paris-Dauphine, PSL Research University, Place du Maréchal de Lattre de Tassigny, 75016 Paris, France

**Keywords:** gender stereotypes, gender equality, cross-cultural variations, gender equality paradox, word embeddings

## Abstract

Gender stereotypes contribute to gender imbalances, and analyzing their variations across countries is important for understanding and mitigating gender inequalities. However, measuring stereotypes is difficult, particularly in a cross-cultural context. Word embeddings are a recent useful tool in natural language processing permitting to measure the collective gender stereotypes embedded in a society. In this work, we used word embedding models pre-trained on large text corpora from more than 70 different countries to examine how gender stereotypes vary across countries. We considered stereotypes associating men with career and women with family as well as those associating men with math or science and women with arts or liberal arts. Relying on two different sources (Wikipedia and Common Crawl), we found that these gender stereotypes are all significantly more pronounced in the text corpora of more economically developed and more individualistic countries. Our analysis suggests that more economically developed countries, while being more gender equal along several dimensions, also have stronger gender stereotypes. Public policy aiming at mitigating gender imbalances in these countries should take this feature into account. Besides, our analysis sheds light on the “gender equality paradox,” i.e. on the fact that gender imbalances in a large number of domains are paradoxically stronger in more developed/gender equal/individualistic countries.

**Significance Statement**

Gender stereotypes are related to gender imbalances, and their analysis, in particular in a cross-country context, is important. Gender stereotypes have cultural foundations, and we expect them to vary across countries and to be reduced in more economically developed countries. They are however difficult to measure. Leveraging a recent tool in natural language processing, we are able to analyze the variations across countries of gender stereotypes embedded in natural language. We find that gender stereotypes about career, math, or science are all stronger in the text corpora of more economically developed and individualistic countries. Our results suggest that these countries, while being more gender equal in several domains, also have stronger gender stereotypes.

## Introduction

Gender stereotypes have deep influences on how men and women are perceived and perceive themselves, on their attitudes and preferences and on their choices (1–4). Common gender stereotypes include the association of men with professional life and women with domestic life, as well as the association of men with math or science and women with arts or liberal arts. These biased associations act as an unseen force steering men and women to different behaviors, roles, and activities and contribute to gender imbalances at home, at school, in educational choices, and in the labor market. Having cultural foundations, gender stereotypes should vary in strength across countries, and an analysis of their variations across countries can help us better understand and reduce gender imbalances.

Besides, an analysis of gender stereotypes in a cross-country context could shed some light on the gender equality (or development) paradox. This paradox is the now well-replicated fact that gender differences in a large number of domains are not reduced but greater in countries with higher levels of economic development, individualism, or gender equality. First results were provided by the large-scale study (5) showing that gender differences in personality traits were larger in more individualistic and affluent countries. Similar results were subsequently obtained for gender differences in values (6), preferences (7), tastes (8), or choices of occupations (9, 10). Possible explanations for the gender equality/development paradox include, among others, the existence of innate gender differences that can be more easily expressed under more favorable social and economic conditions (10, 11) or the presence of gender stereotypes that could be more prevalent and readily expressed in individualistic and developed countries (9, 12–15), these lines of explanations being not mutually exclusive. An analysis of the cross-country variations of gender stereotypes, and in particular of the variations of gender stereotypes with indicators of country economic development, individualism, and gender equality, would help to better identify the possible role of stereotypes in the gender equality/development paradox and more generally to gain a deeper understanding of the paradox.

Gender stereotypes are difficult to measure, especially in a cross-county context. Word embeddings are a recent natural language processing (NLP) tool that has shown validity and reliability in measuring gender stereotypes, even in a cross-country context (16–21). Word embeddings are numeric representations of meaning derived from word cooccurrence statistics in corpora of human-produced texts. By capturing semantic similarities between words of the corpora on which they are trained, word embeddings can serve as a powerful tool to detect gender associations at a societal level of analysis on a large scale.

In this contribution, we attempt to analyze cross-country variations in gender stereotypes by relying on publicly available embedding models pretrained on text corpora from more than 70 countries. We consider gender stereotypes that have been robustly documented in previous literature on both explicit and implicit measures. Specifically, we examine the stereotyped male–career/female–family association (22), as well as the male–math/female–liberal arts (23) and the male–science/female–arts associations [for a review, see (24)].

We expect that these stereotypes will vary in strength across countries but the way they should vary with country level of economic development, individualism, or gender equality is not clear. First, lay expectations suggest that gender stereotypes should be less pronounced in such countries, where there are fewer disparities in “vertical” opportunities such as educational attainment and labor force participation and where men and women are considered and treated more equally due to prevailing gender egalitarian values. In support of these arguments, Inglehart and Norris suggested in their “rising tide” theory (25) that development induces systematic changes in gender roles toward greater gender equality in any society. More recently, higher levels of gender equality at the country level were shown to be associated with reduced male dominance in Google image search results for the gender neutral keyword “person” (26). However, the gender equality/development paradox reveals strong gender imbalances in traits, values, or “horizontal” opportunities in more economically developed countries (5, 6, 9, 10). These imbalances could be associated to the presence of stronger gender stereotypes in these countries, either as a cause (stereotypes influencing gender imbalances) or as a consequence (stereotypes reflecting gender imbalances). In line with this argument, Williams and Best's seminal international study on gender stereotypes in personality traits across 26 countries reveals that men and women are perceived as relatively more different in more developed and individualistic countries [(27), p. 27]. A recent study (12) uses students’ questionnaires in the Program for International Student Assessment (PISA2012) to propose a “nonstandard” measure of the stereotype that math is not for girls and shows that this stereotype is more prevalent in more developed countries. Could the same pattern be observed for gender stereotypes about career, math, and science embedded in natural language?

Our findings show that gender stereotypes about career, math, and science indeed vary across countries and are all stronger in the text corpora of more economically developed and individualistic countries. Gender stereotypes embedded in natural language are also stronger in more gender equal countries, in particular in countries that endorse more gender egalitarian values, but the relation seems weaker than with countries’ wealth or individualism. We discuss the possible mechanisms connecting economic development or individualism to gender stereotypes by drawing upon previous research in sociology that emphasizes the multidimensional nature of gender equality (2, 13, 28–33) and allows us to suggest possible explanations for the fact that in more wealthy and individualistic countries, men and women can be considered as both more equal and more different. Regardless of the origin of these stronger gender stereotypes, their prevalence should be noted since they are likely to impact gender imbalances. We finally discuss the implications of our results as well as their limitations. In particular, we emphasize the importance of exercising caution when interpreting our results, as they are based on big data analysis in an international context and may involve various underlying mechanisms.

## Results

### Descriptive statistics

Our approach is similar to the one in (16) and detailed in the “Materials and methods” section and in Appendix A. We summarize the main steps here. As in (17), we rely on publicly available word embeddings pretrained on text corpora from Wikipedia (34) and from the Common Crawl Project (35), using the fastText algorithm [a variant of word2vec (36)]. The Wikipedia and Common Crawl corpora are organized and split by language, and our sample of text corpora includes 82 language corpora from Wikipedia and 75 language corpora from Common Crawl. We consider linguistic stereotypical gender associations about career–family, math–liberal arts, and science–arts. For each stereotype, we choose stimuli, i.e. sets of words representing the categories men and women as well as sets of words representing the attributes career–family, math–liberal arts, and science–arts. We essentially adopt the same stimuli as in (16) for the English corpus, which are the stimuli of the implicit association test (IAT), and translate these stimuli for all other language corpora. As in (16), our measures of gender stereotype, denoted by GS, rely on the word embedding association test (WEAT), which tweaks the IAT for word embeddings. For each corpus, the measures GS represent the extent to which male words are more similar than female words to career versus family words, to math versus liberal arts words, and to science versus arts words. We also consider a measure of aggregate gender stereotypes by collapsing the three stereotypes (career, math, and science, see the “Materials and methods” section).

Tables S1 and S2 provide the three gender stereotypes measures GS by language corpus, as well as the effect sizes (i.e. normalized measures of gender stereotypes, see the “Materials and methods” section). The average effect size varies from 0.35 for career–family associations to 0.57 for math–liberal arts associations (for Wikipedia). The measures of stereotypes are positive in most corpora, reflecting biased associations in favor of males for career, math, and science, but there is variation in the level of biased associations across corpora (e.g. SD = 0.53 for math–liberal arts effect sizes) and there are language corpora where the association is biased in favor of females. Figure S1 provides the histograms of the three GS (Wikipedia).

We analyze in Table 1 the relations between GS measures across corpus sources (Wikipedia and Common Crawl) and across stereotypes (career, math, and science). Concerning corpus sources, the correlation between the measure of aggregate stereotype from Wikipedia and that from Common Crawl is significant but not perfect with *R* = 0.59. Figure S2 illustrates this result. For the three stereotypes taken individually, we get *R* = 0.66 for career–family GS, *R* = 0.49 for math–liberal arts GS, and *R* = 0.42 for science–arts GS. GS measures are consistent but slightly different across sources, plausibly due to the variations in content, Wikipedia being more specific, and considered more objective and fact based (17, 37, 38). The correlation across different stereotypes is also significant, with *R* = 0.52 between career and math stereotypes, *R* = 0.51 between career and science stereotypes, and *R* = 0.75 between math and science stereotypes (for Wikipedia). This means that corpora that exhibit stronger biased gender associations of one type (e.g. career–family stereotypes) are also those that exhibit biased gender associations of the other types (stronger math–liberal arts and science–arts stereotypes). These corpora are altogether more gender stereotyped.

**Table 1.** Relations of linguistic gender stereotypes across corpus sources (wikipedia and common crawl) and across stereotypes (career–family, math–liberal arts, and science–arts). Relations with country levels of individualism and gross national income.

	Career–family WIKI	Career–family CC	Math–liberal arts WIKI	Math–liberal arts CC	Science–arts WIKI	Science–arts CC	All WIKI	All CC
Career Wiki	1							
Career CC	0.655^a^	1						
Math Wiki	0.518^a^	0.146	1					
Math CC	0.228^b^	0,243^b^	0.490^a^	1				
Science Wiki	0.510^a^	0.216^c^	0.753^a^	0.338^a^	1			
Science CC	0.330^a^	0.377^a^	0.257^b^	0.466^a^	0.423^a^	1		
All Wiki	0.926^a^	0.529^a^	0.775^a^	0.352^a^	0.768^a^	0.383^a^	1	
All CC	0.637^a^	0.913^a^	0.303^a^	0.563^a^	0.357^a^	0.653^a^	0.587^a^	1
Indiv.	0.558^a^	0.598^a^	0.318^a^	0.365^a^	0.408^a^	0.386^a^	0.565^a^	0.660^a^
GNI	0.471^a^	0.444^a^	0.283^b^	0.260^b^	0.273^b^	0.311^b^	0.465^a^	0.493^a^

Presents estimates of correlation coefficients among gender stereotypes embedded in Wikipedia corpora (Wiki, columns 1, 3, 5, and 7), gender stereotypes embedded in Common Crawl corpora (CC, columns 2, 4, 6, and 8), and country-level measures of individualism (Hofstede, Indiv.) and economic development (GNI). We consider gender stereotypes about career and family (columns 1 and 2), about math and liberal arts (columns 3 and 4), and about science and arts (columns 5 and 6). Measures of gender stereotypes rely on semantic similarity between male and female words and words about career–family, math–liberal arts, and science–arts. We also consider a measure of aggregate gender stereotype, which is a weighted average of the three gender stereotypes (columns 7 and 8). For the relations with individualism and economic development, we restrict the samples to countries with available data for both Hofstede's measure and GNI. Details about methods and data are provided in the Supplementary material.

^a^ *P < 0.01.* ^b^ *P < 0.05.* ^c^ *P < 0.1*.

### Gender stereotypes are stronger in the text corpora of more economically developed and individualistic countries

We now examine how gender stereotypes GS vary across countries and in particular how they vary with levels of economic development and individualism. We use gross national income (GNI) to measure economic development and Hofstede's index of individualism versus collectivism to measure individualism. Since these indices are calculated per country and gender stereotypes are available by language corpus (and not per country), we followed the approach of (17) and used data from Wikipedia, which outlines the relative contribution of residents from each country to each language corpus (Wikimedia Foundation^1^). In our main specification, we consider for each language corpus the (level of economic development and of individualism of the) country that contributes the most to the corpus (see details in the “Materials and methods” section and Supplementary material). This amounts to assuming that the stereotypes embedded in a corpus reflect the stereotypes of the residents of the country that contributes the most to the corpus. As robustness checks, we consider alternative ways to match corpora with levels of economic development and individualism, in particular in proportion to country's relative contribution to the corpus as in (17).

We first observe that the level of aggregate gender stereotypes is on average twice as large in the text corpora of OECD (Organization for Economic Co-Operation and Development) countries, which are mostly economically developed countries, as in the corpora of non-OECD countries (Table S3).

**Fig. 1.**
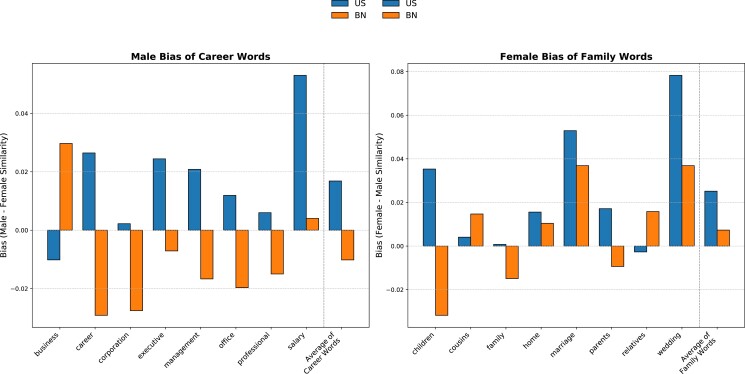
Linguistic gender stereotypes about career and family embedded in the US and Bangladesh Common Crawl text corpora. The figure presents measures of the relative semantic similarity of career and family words to male versus female words in the US (US, English language) and in the Bangladesh (BN, Bengali language) Common Crawl text corpora. The words stimuli to represent career and family attributes are the stimuli used in the IAT and the same as in (10) and (1), i.e. eight career-related words: “business,” “career,” “corporation,” “executive,” “management,” “office,” “professional,” and “salary” and eight family-related words: “children,” “cousins,” “family,” “home,” “marriage,” “parents,” “relatives,” and “wedding.” The left figure represents the relative level of similarity of career words to male words versus female words (for each word separately and on average in the last column). Positive values represent a male bias, i.e. a greater similarity to male words than female words. The right figure represents the relative level of similarity of family words to female words versus male words (for each word separately, and on average in the last column). Positive values correspond to a female bias. Details about methods and data are provided in the “Materials and methods” section and in the Supplementary material.

Figure 1 provides a comparative analysis of career–family stereotypes embedded in the (Common Crawl) text corpora of two countries: an OECD country, known for its economic development and individualism (the United States), and a non-OECD country, characterized by lower economic development and individualism (Bangladesh). We observe distinct patterns in the semantic similarity of career and family words to male versus female words in these two corpora. Specifically, in the US corpus, all career-related words except “business” show a stronger semantic similarity to male words than female words, with the strongest male biases observed for the words “career” and “salary.” In contrast, the Bangladesh corpus displays more balanced semantic similarities to male and female words for career-related words, resulting in a slight female bias. Turning to family-related words, a similar trend emerges. In the US corpus, all family-related words except “relatives” exhibit a stronger semantic similarity to female words than male words, with “wedding” and “children” showing particularly strong female biases, whereas the similarities to male and female words are more balanced in the Bangladesh corpus. As a consequence, the career–family gender stereotype is notably more pronounced in the US corpus (GS = 0.042) than in the Bangladesh corpus (GS = −0.003; see Table S1).

**Fig. 2.**
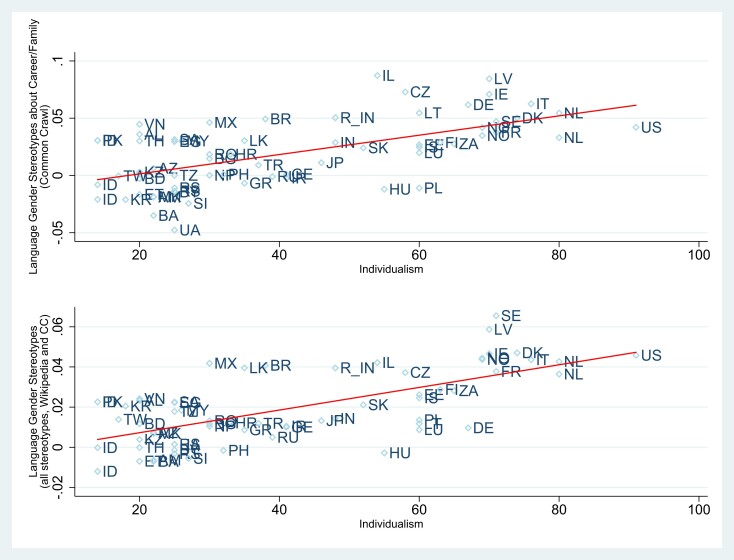
Gender stereotypes embedded in natural language as a function of country level of individualism. This presents measures of language gender stereotypes as a function of country level of individualism (Hofstede's measure of individualism). The top figure considers gender stereotypes about career and family embedded in the text corpora of the Common Crawl project, while the bottom figure considers the three gender stereotypes about career, math, and science embedded in text corpora from both sources, Wikipedia and Common Crawl. Gender stereotypes are measured through the Word Embedding Association Test (16). Details about methods and data are provided in the Supplementary material.

More precisely, Table 1 and 2 and Table S3 provide the relation between our GS measures and country level of GNI and individualism. For the Wikipedia (Common Crawl) source, a 1 SD increase in country level of individualism is associated with an increase of 0.56 SD (0.60 SD) of the level of career–family GS and a 1 SD increase in country level of GNI is associated with an increase of 0.47 SD (0.44 SD) of career–family GS. Figure 2 and Figure S3 illustrate this result. For math and science, the increases in the level of GS associated with a 1 SD increase in country level of individualism or GNI vary between 0.26 SD and 0.41 SD and are all significant. If we consider the three stereotypes together, we find that a 1 SD increase in country level of individualism (resp. GNI) is associated with an increase of 0.66SD (resp. 0.49SD) of the level of aggregate GS in the Common Crawl corpus source. Collapsing both sources, we obtain in Table 2 that a 1 SD increase in country level of individualism (resp. GNI) is associated with an increase of 0.67 SD (resp. 0.54 SD) of the level of aggregate GS. Figure 2 illustrates this result. Finally, Table S3D suggests that the impact of individualism seems stronger than the impact of GNI on aggregate gender stereotypes GS, in the sense that if both indices are introduced simultaneously as explanatory variables in the regressions, only the index of individualism remains significant (and barely altered).

**Table 2.** Regression of linguistic gender stereotypes about career–family, math–liberal arts, and science–arts on country individualism and gross national income.

	Dependent variable is language gender stereotype about							
	Career–family, math–liberal arts, science–arts	Career–family	Math–liberal arts	Science–arts	Career–family, math–liberal arts, science–arts	Career–family	Math–liberal arts	Science–arts
Individualism	0.665^a^ (0.0956)	0.630^a^ (0.0994)	0.372^a^ (0.119)	0.462^a^ (0.114)				
GNI					0.538^a^ (0.108)	0.507^a^ (0.110)	0.297^b^ (0.122)	0.389^a^ (0.118)
Constant	1.17e−10 (0.0948)	3.00e−09 (0.0986)	−2.59e−09 (0.118)	−5.29e−09 (0.113)	−2.75e−10 (0.107)	2.63e−09 (0.109)	−2.81e−09 (0.121)	−5.56e−09 (0.117)
Observations	63	63	63	63	63	63	63	63
*R* ^2^	0.443	0.397	0.138	0.214	0.290	0.257	0.088	0.151

Presents estimates of linear regressions of country-level gender stereotypes (based on semantic similarity in both Wikipedia and Common Crawl corpora) on country levels of individualism (Hofstede's measure of individualism) and economic development (GNI). Language gender stereotypes are about career, math, and science and measured as in (16) by the Word Embedding Association Test. Columns 2–4 and 6–8 consider each stereotype separately: male–career/female–family stereotypes in columns 2 and 6, male–math/female–liberal arts stereotypes in columns 3 and 7, and male–science/female–arts stereotypes in columns 4 and 8. Columns 1 and 5 consider the three stereotypes together. The sample is restricted to countries with available data for Hofstede's measure of individualism and GNI, and all variables are standardized on the regression sample. SEs in parentheses.

^a^ *P < 0.01.* ^b^ *P < 0.05*. ^c^*P < 0.1*.

### Single-category biased associations

As the standard IAT, the WEAT measure, hence our GS measure, makes it impossible to disentangle the various associations and to determine whether the stronger gender stereotypes in more individualistic and economically developed countries are primarily related to male or to female words and whether they are primarily related to a stronger relative similarity of career/math/science words to male versus female words or to a stronger relative similarity of family/liberal arts/arts words to female versus male words. As the single category IAT (39), the single category WEAT introduced in (19, 40) (see the “Materials and methods” section and the Supplementary material) makes it possible to quantify each association separately.

Table S4 shows first that the relation between GS and country individualism and GNI is primarily related to female words (relative to male words). A 1 SD increase in the level of individualism (resp. GNI) is associated with a decrease of 0.43 SD (resp. 0.25 SD) in the similarity of female words to career versus family words (Wikipedia). Figure S4 illustrates this result. The relation for male words is not significant. The pattern is robust to the consideration of aggregate stereotypes and of the Common Crawl source (Tables S4C and S4D).

Concerning attributes, Table S4 shows that career/math/science words are more similar to male words versus female words in more individualistic and economically developed countries. A 1 SD increase in the level of individualism (resp. GNI) is associated with an increase of 0.44 SD (resp. 0.39 SD) in the similarity of career/math/science words to male versus female words (Wikipedia). Figures S4 and S5 illustrate the result. Table S4 further shows that there is a lower association of family words to male versus female words in more individualistic and economically developed countries for the Wikipedia corpora. This relation is however not robust to the consideration of the Common Crawl corpora (Table S4D).

### Robustness checks and complementary analyses

#### Robustness to other stimuli

Since our analysis relies on word embeddings and word similarity, the choice of the stimuli is important, and we verify the robustness of our results to alternative stimuli. We consider as alternative stimuli for the career–family dimension and for the science–arts dimension the stimuli adopted in (40) for these dimensions (see the “Materials and methods” section as well as Appendix A for the detailed sets of words). Table S5A provides descriptive statistics (for Wikipedia). The average GS computed for these alternative stimuli is very similar to the average GS in our main analysis, for both stereotypes (0.026 vs 0.024 for career–family and 0.016 vs 0.015 for science–arts). Table S5B shows that for both dimensions, the correlation between our main measure and the measure obtained with the alternative stimuli is strong, with *R* = 0.80 for the career–family dimension and *R* = 0.74 for the science–arts dimension (Wikipedia). Table S5C shows that the main result of our analysis remains valid with these alternative stimuli, a 1 SD increase in country level of individualism (resp. GNI) being associated with an increase of 0.35 SD (resp. 0.34 SD) of career–family GS and of 0.38 SD (resp. 0.35 SD) of science–art GS (for Wikipedia).

#### Robustness to other measures of individualism and economic development

We have considered so far GNI as a measure of economic development and Hofstede's index of individualism–collectivism as a measure of individualism. We verify the robustness of our conclusions to alternative measures of country individualism and economic development. We consider as an alternative measure of individualism the opposite of the measure of collectivism of the Globe survey, which is more related to *family* collectivism than the measure of Hofstede (41). As alternative measures of country economic development, we consider the gross domestic product (GDP), as well as the human development index (HDI) which incorporates measures of education and life expectancy on top of economic wealth. The definition of these measures and data sources are provided in Appendix B. Table S6A shows that a 1 SD increase in these various measures of individualism and economic development is associated with an increase of aggregate gender stereotypes (collapsed across corpus sources) of 0.38 SD for HDI, 0.48 SD for GDP, and 0.56 SD for the opposite of the collectivism measure of the Globe survey. Similar results are obtained when considering each stereotype and each source of text corpora separately (Table S6A).

Table S6B shows that our results are fully robust to the consideration of historical measures of economic development (like GDP in 1960 and 1970). The number of observations is sometimes reduced for these historical measures, but Table S6 shows a systematic significant relation with gender stereotypes, taken individually or collapsed into an aggregate measure of stereotypes, which indicates that the relation between stereotypes and country levels of individualism and wealth is historically founded.

#### Consideration of country gender equality and ecological stress

We have focused so far on country individualism and economic development because these dimensions are considered as main drivers of cultural variations and because they are the most frequently considered indices in the literature related to the “gender-equality/development paradox.” Numerous works, however, have related the gender equality paradox directly to the level of gender equality of the countries (10) or to their level of ecological stress (42), and we now examine the relation between gender stereotypes GS and these constructs.

Table S7A first shows that the relation is significant in most specifications for the gender gap index (GGI), which synthesizes the position of women regarding education, economic opportunities, politics, and health, and has been shown in (10) to be related to sex differences in STEM (Science, Technology, Engineering and Math) fields. A 1 SD increase in the GGI is associated with an increase of aggregate GS embedded in both data sources of 0.3 SD. This means that gender stereotypes embedded in languages are stronger in environments that are more gender equal in the sense of the GGI. Since the GGI is a composite index, it is useful to consider specific dimensions of gender equality. The relation is not robustly significant with measures of gender equality (in practice) that are specifically related to career or family issues, like female fertility rate or female labor force participation (see data sources in the Supplementary material). The relation is, on the contrary, strong and robustly significant with measures of gender equality in values. A 1 SD increase in the level of gender equality in values concerning politics, education, or occupations is associated with an increase of aggregate GS embedded in both data sources between 0.52 SD and 0.62 SD (Table S7A). The relation of gender stereotypes with GGI or measures of gender equality in values is however weaker than with measures of individualism or GNI, in the sense that the relation mostly disappears when controlling for either of these measures (Table S8A and B). Note that for stereotypes about math, the relation with the GGI, and especially with gender equality in terms of values, is more robust when controlling for GNI or individualism compared with the other stereotypes, which suggests that the impact of (some measures of) gender equality per se is greater on math-related stereotypes than on those related to career or science.

Ecological stress has been shown to be related to collectivism (43) and also to sex differences in, e.g., personality traits (42). In (42), when controlling for confounds (like various measures of gender equality and of economic development), only the impact of ecological stress and of individualism remained. As in (42), we consider the two most prominent sources of ecological stress, i.e. lack of nutrition and disease prevalence, using both historical and contemporary estimates (see Supplementary material). Table S7B shows that the relation is significant in most specifications. A 1 SD increase in the level of lack of nutrition is, for instance, associated with a decrease of aggregate GS embedded in both data sources of 0.48 SD for contemporary estimates and 0.39 SD for historical estimates. For both sources of ecological stress, the relation seems however to disappear when controlling for the levels of country individualism or GNI (Table S8C).

In summary, gender stereotypes are stronger in the text corpora of more gender equal countries (in the sense of the GGI or gender equality in values) or countries facing lower ecological stress, but these relations seem to be weaker than those observed with country level of individualism or GNI.

#### Additional robustness checks

Table S9 shows that results remain the same when considering *normalized* measures of gender stereotypes, i.e. effect sizes (see Supplementary material). Results also remain qualitatively and quantitatively valid if we restrict our sample of text corpora (i) to the largest contributors to the Wikipedia data source (Table S10, column 4, see Supplementary material) or (ii) to OECD countries that are mostly economically developed countries (Table S10, column 5). DeFranza *et al.* (17) have shown the relation between genderedness of languages and the strength of gender prejudice. Since individualism and economic development may be related to the genderedness of languages, we verify if our results remain valid when controlling for genderedness. Table S10 (column 6) shows that it is indeed the case. Table S10 further shows that results are robust in controlling for a metric of country-level interdependence, such as continent and language family (see Supplementary material).

## Discussion

Relying on word embeddings from two different data sources (Wikipedia and Common Crawl), each comprising around 80 text corpora, we have found that gender stereotypes embedded in natural language are stronger in the corpora of more individualistic and economically developed countries. Gender stereotypes are also stronger in the corpora of more gender equal countries (in the sense of the GGI or of gender egalitarian values), although the relation seems weaker than with individualism or economic development. These results hold true for gender stereotypes about career–family, math–liberal arts, and science–arts. In this discussion, we aim to provide an interpretation of our findings, explore their implications, and acknowledge the limitations of our approach.

### Interpretation of our findings

We interpret our result as reflecting the presence of stronger gender stereotypes (about career, math, and science) in more economically developed and individualistic countries, which may initially seem surprising, as mentioned in the “Introduction” section.

This finding is however in line with the few previous empirical works on the variations of gender stereotypes across countries (12, 15, 27).

Identifying the exact mechanism leading to stronger gender stereotypes in (the natural language of) more developed, individualistic, or gender equal countries is beyond the scope of this work, but there are theoretical foundations to both interpret and explain why in these countries higher formal procedural equality can coexist with stronger stereotypes of gender differences.

Prominent theories of social norms propose that dominant social groups utilize norms to distinguish themselves (44). They highlight how eliminated sources of social differentiation are likely to be replaced by other type of norms to sustain social hierarchy. This can explain why in more individualistic and progressive countries, in which male primacy is reduced, an “equal but different” ideology can be reinforced. Moreover, according to social dominance theory, the most culturally valued traits in a society are attributed to more dominant social groups, which are usually men (45, 46). Thus, stereotypes about men and women will change with the core values of a given culture, which can explain, for instance, why in more individualistic and progressive countries, in which individualism is more valued, career (resp. family) is relatively more associated with men (resp. women) (47).

This interpretation is also supported by recent research in sociology on gender norms that emphasizes the role of gender as a fundamental cultural tool for framing social relations and the resistance to any real reduction in gender differentiation (2, 4, 13, 28–33). This research argues that gender differentiation is maintained and rewritten into new socioeconomic arrangements, even under an altered form (4). In particular, it underscores the significance of distinguishing between two dimensions of gender ideology: the belief that men and women are unequal (related to vertical differentiation) and the belief that they are on balance different (related to horizontal differentiation) (2, 13, 28–33). The former has declined in developed and individualistic countries, but it has been replaced by various forms of egalitarianism, with the emergence of a new frame, denoted “egalitarian essentialism” (2) combining support for traditional differentiation between men and women with an individualistic and feminist rhetoric of choice and equality (28, 29). It denies implications of lower status for women and can claim to be egalitarian since men and women have equal opportunities and can make counter-stereotypical choices if they want to, while still reinforcing traditional gender stereotypes of complementarity.

Research on the gender equality/development paradox also provides interesting insights into the factors that can contribute to the endorsement of stronger gender stereotypes in individualistic and wealthy countries. First, the impact of individualism and economic development on gender stereotypes can be indirect, acting through strong existing gender imbalances within these countries. Indeed, the gender equality paradox highlights the presence of strong gender differences in preferences, behaviors, and choices in individualistic and wealthy countries. These differences may arise because men and women can better express their innate preferences (based on evolutionary arguments) or due to variations in socioeconomic and cultural environments. Therefore, the strong biased gender associations observed in the natural language of these countries may simply be the reflection of these strong existing gender disparities in preferences, behaviors, and choices. However, individualism and economic development may impact gender stereotypes more directly. First, as noted by Charles and Bradley (9), individualistic and wealthy societies emphasize the importance of the individual, highlighting individual personality traits and preferences as well as the idea of an individual's “true self.” As underlined in (9), these notions of self are in fact largely socially constructed drawing from traditional gender stereotypes, which can lead to the reinforcement of such stereotypes in individualistic societies. Another, and perhaps most plausible, explanation relies on attribution processes (48, 49), as suggested by Costa *et al.* (5). The idea is that in individualistic and wealthy countries, where men and women are considered and treated more similarly, the fact that women spend more time with their children than men, or neglect math fields, may be seen as a free choice, reflecting their preferences, whereas the same behaviors in a more collectivistic and traditional country might be seen as mere compliance with sex role norms and requirements. Differences in behaviors will then be attributed to differences in preferences and traits rather than constraints and roles, which can contribute to the reinforcement of standard gender stereotypes in more individualistic and progressive societies.

We emphasize that these explanations are not mutually exclusive and can complement one another to contribute to the reinforcement of stereotypes in wealthy and individualistic countries.

### Implications of our analysis

Whatever the underlying mechanism, our analysis highlights the presence of stronger gender stereotypes in the natural language of more individualistic and wealthy countries, which has both practical and theoretical implications.

Our analysis first emphasizes the importance of trying to reduce gender stereotypes in wealthy and individualistic countries or at least to minimize their impact or to be aware of their presence to allow more gender equality. This is true of stereotypes embedded in language, but more generally of stereotypes embedded in society, that the word embeddings reflect. This would be beneficial to both women and men. Directly attacking gender stereotypes is difficult, but being aware of the presence of strong gender stereotypes in developed countries is important when considering possible gender equality policies. For example, easier or longer paternity or maternity leave may backfire and reinforce labor market imbalances if, due to the presence of strong stereotypes, women make more use of it than men or if fathers use it for purposes other than childcare.

Our analysis also has theoretical implications. It shows that in more individualistic and economically developed countries, more gender egalitarian values and practices can coexist with stronger gender stereotypes, which supports the growing literature underlining the multidimensional nature of gender equality (2, 9, 28, 30, 50).

Besides, our analysis provides new insights into the gender equality paradox. It does not invalidate existing explanations, and these explanations probably complement one another. However, in line with (9, 12), our analysis suggests that stereotypes are likely part of the explanation, as regardless of their origin, the mere presence of more pronounced stereotypes in (the natural language of) individualistic and developed countries should lead to greater gender imbalances (in traits, in values, in preferences, in choices). This is all the more true as more developed and individualistic countries make the expression of gender stereotypes into “free” choices easier (9). More generally, existing and future possible explanations of the paradox should be analyzed in terms of their consistency with the fact that gender stereotypes themselves seem more marked in more developed countries.

A final implication is directly related to our method of measuring stereotypes through word embeddings. The fact that word embeddings trained on Wikipedia and Common Crawl corpora exhibit gender stereotypes in wealthy and individualistic countries is concerning because, as underlined by Bolukbasi *et al.* (21), due to their wide-spread usage as basic features, word embeddings not only reflect gender stereotypes present in broader society but can also perpetuate and amplify these stereotypes. This is particularly worrying with their widespread use in NLP applications such as (hiring) decision tools or the ongoing development of generative artificial intelligence.

### Limitations of our analysis

First, our analysis is correlational. It shows that individualistic and wealthy environments are associated with stronger gender stereotypes embedded in language, but cannot make any claim about causality, even if we have provided possible explanations. However, note that whatever the cause of these stereotypes, it is important to identify the presence of strong gender stereotypes in (the text corpora of) individualistic and wealthy countries to better understand and reduce gender imbalances.

Second, we relied on word embeddings to measure gender stereotypes, and while word embeddings have been shown to be a reliable method to detect biased associations and cultural beliefs in a society, they are not without limitations.

In particular, their capture of gender stereotypes may be noisy, especially in a cross-country context. The measure of biased associations may be influenced by corpus selection and specific stimuli choices. Additionally, due to diverse languages in the corpora, we had to rely on a translation tool, introducing some noise, even if we have confirmed the robustness of the results to various choices of corpora, stimuli, and translation tools.

Regarding the relation with macro variables, each corpus does not univocally correspond to a single country, and we had to choose a procedure to associate a level of individualism and wealth with each text corpus (see “Materials and methods” section), which may also introduce noise, even if we verified the robustness to different matching procedures. Moreover, the volume of Wikipedia or Common Crawl data can be influenced by factors like internet usage, which is related to economic development. Similarly, the volume of data related to gender or related to career/math/science in a country can be influenced by economic development and impact the measure of gender stereotypes, even if the nature of this impact is not clear. Gender stereotypes themselves may be more openly discussed in Western countries, and there may be a greater volume of research in social psychology there, which could affect the dataset. Furthermore, specific linguistic features that may vary with country's level of individualism or wealth could potentially introduce bias into the measurement of gender associations.

All of these limitations emphasize the need for careful interpretation of the results based on word embeddings, and in future research, it would be valuable to assess the robustness of our analysis using alternative measures of gender stereotypes.

Third, we have focused on gender stereotypes that have been robustly documented in the literature, namely stereotypes about career/family and about math or science/arts or liberal arts. While these are common gender stereotypes in Western countries, there may exist other stereotypes, like e.g. stereotypes more directly related to a gender hierarchy, that could be stronger in less wealthy and individualistic countries. For instance, Bailey *et al.* (51) have recently shown, by using word embeddings trained on the Common Crawl corpus, that the concept person/people prioritizes men over women, and this biased association could be stronger in less affluent and individualistic countries. Note however that these stereotypes would be less directly related to the gender imbalances that satisfy the gender equality paradox that are mainly “horizontal.”

Finally, word embeddings relying on large-scale corpora such as Wikipedia or Common Crawl make it impossible to distinguish between stereotypes endorsed by men and stereotypes endorsed by women. Such a distinction can be useful, in particular to examine the relationship between gender stereotypes and gender differences in individual choices, and could be made by relying on other measures of stereotypes, such as the IAT, or by relying on word embeddings trained on other text corpora, such as books [as in (52)] or song lyrics [as in (53)].

## Materials and methods

### Word embeddings and text corpora

We used publicly available word embeddings, pretrained using the fastText algorithm on two different data sources, with each more than 100 text corpora corresponding to different languages. The first data source consisted of data from the free online encyclopedia Wikipedia (34), and the second was from the Common Crawl project, which contains snapshots of all the text that can be scraped from the publicly facing Internet, since 2013 (35). We kept the text corpora (i) whose language is an official language of at least one country and (ii) for which the contribution of each country is available (Wikimedia Foundation). See the Supplementary material for more details. Our final sample included 82 corpora for the Wikipedia project and 75 corpora for the Common Crawl Project.

### Word embedding association test

We used the WEAT (16) to obtain measures of gender stereotypes from word embeddings. Tweaking the IAT, the WEAT measures the association between two sets of target words *X* and *Y* (e.g. males and females) and two sets of attribute words *A* and *B* (e.g. career and family). The WEAT measures the difference between the two sets of target words in terms of their relative similarity to the two sets of attribute words.

The level of similarity between two words $w_{1}$ and $w_{2}$ with word embeddings $\vec{w_{1}}$ and $\vec{w_{2}}$ is defined as the cosine similarity $\cos\;(\vec{w_{1}},\vec{w_{2}})=\vec{w_{1}}\cdot \vec{w_{2}}/∥\vec{w_{1}}∥∥\vec{w_{2}}∥$, where the numerator represents the inner product of the embeddings $\vec{w_{1}}$ and $\vec{w_{2}}$, while the denominator is the product of their respective norms. For an individual target word *w*, its relative level of similarity to the set of words *A* versus the set *B* is given by $s(w,A,B)=\mathrm{mea}n_{a\in A}\cos\;(\vec{w},\vec{a})-\mathrm{mea}n_{b\in B}\cos\;(\vec{w},\vec{b})$. The relative similarity of the target set *X* to the attribute set *A* versus the set *B* is then given by $s(X;A,B)=\mathrm{mea}n_{x\in X}s(x,A,B)$ and the relative similarity of the target set *Y* by $s(Y;A,B)=\mathrm{mea}n_{y\in Y}s(y,A,B)$. The level of association between the sets of target words (X,Y) and the sets of attribute words (A,B) is given by the difference s(X,Y;A,B)=s(X;A,B)−s(Y;A,B). We also consider a normalized level of association or effect size defined by $\mathrm{ES}(X,Y;A,B)=s(X,Y;A,B)/SD_{w\in X\cup Y}s(w,A,B)$.

### Measures GS of language gender stereotypes

To measure gender stereotypes, we consider as sets of target words, a set *X* of male words and a set *Y* of female words. The sets of attribute words change depending on the gender stereotype under consideration, with a set *A* of career words (resp. math, science words) and a set *B* of family words (resp. liberal arts, arts words) for gender stereotypes about career-family (resp. math–liberal arts, science–arts). For each stereotype represented by the sets (A,B), we take as our measure of gender stereotypes GS(A,B)≡s(X,Y;A,B). Higher values of GS(A,B) correspond to stronger gender stereotypes (A,B). For example, for gender stereotypes about career–family, then *A* is a set of career words, *B* is a set of family words, s(X;A,B)—respectively s(Y;A,B)—measures the relative higher similarity of male words—respectively female words—to career versus family words, and GS(A,B) measures the differential similarity between male and female words to career versus family words, i.e. the level of gender stereotypes about career–family. Note that unlike the normalized measure ES(A,B), the measure GS(A,B) is linear and permits to easily collapse different gender stereotypes or data sources since $\mathrm{GS}(A\cup A^{\prime },B\cup B^{\prime })=[\mathrm{GS}(A,B)+\mathrm{GS}(A^{\prime },B^{\prime })]/2$, and this is one of the reasons why we favor this measure in our analysis. In robustness checks (Table S9), we verify that our results remain valid for effect sizes ES(X,Y;A,B).

### Stimuli

Concerning the choice of stimuli (i.e. the sets of words X,Y,A,B), we adopted the same sets as in (16) and as in the IAT for the English text corpus, except that we kept the same sets *X* and *Y* of male and female words for the three different stereotypes. This permits to avoid sets including proper names for which there is no direct translation equivalent in other languages [as in (20)]. More precisely, we took *X* = [“male,” “man,” “boy,” “brother,” “he,” “him,” “his,” “son”] and *Y* = [“female,” “woman,” “girl,” “sister,” “she,” “her,” “hers,” “daughter”] in all settings. For stereotypes about career–family, we took *A* = [“executive,” “management,” “professional,” “corporation,” “salary,” “office,” “business,” “career”] and *B* = [“home,” “parents,” “children,” “family,” “cousins,” “marriage,” “wedding,” “relatives”]. For stereotypes about math–liberal arts, we took *A* = [“math,” “algebra,” “geometry,” “calculus,” “equations,” “computation,” “numbers,” “addition”] and *B* = [“poetry,” “art,” “dance,” “literature,” “novel,” “symphony,” “drama,” “sculpture”], and for stereotypes about science–arts, we took *A* = [“science”, “technology”, “physics,” “chemistry,” “Einstein,” “NASA,” “experiment,” “astronomy”] and *B* = [“poetry,” “art,” “Shakespeare,” “dance,” “literature,” “novel,” “symphony,” “drama”]. In robustness checks, we considered as alternative stimuli those adopted in (40). See Supplementary material for details about these stimuli. For all text corpora in languages other than English, we translated the words in X,Y,A and *B*, from English to the considered language and used these translations to compute gender stereotypes GS(A,B)≡s(X,Y;A,B) embedded in each corpus. In the main specification, we used Google Translate to obtain translation of the words in X,Y,A and *B*, and we verify in Table S11 the robustness of the results to using ChatGPT instead of Google Translate (see also Supplementary material).

### Single-category WEAT

To quantify how “masculine” or “feminine” attribute words are, we define the relative similarity of an attribute word *w* to the target set *X* versus *Y* by $s(w,X,Y)=\mathrm{mea}n_{x\in X}\cos\;(\vec{w},\vec{x})-\mathrm{mea}n_{y\in Y}\cos\;(\vec{w},\vec{y})$, the relative similarity of the attribute set *A* to the set *X* versus the set *Y* by $s(A;X,Y)=\mathrm{mea}n_{a\in A}s(a,X,Y)$, and the relative similarity of the attribute set *B* to the set *X* versus the set *Y* by $s(B;X,Y)=\mathrm{mea}n_{b\in B}s(b,X,Y)$. For instance, for gender stereotypes about career and family, s(A;X,Y) represents the strength of the relative association of career words with male versus female words. Note that for all stereotypes (A,B), our score of gender stereotypes GS(A,B) represents indifferently the difference s(X,Y;A,B)=s(X;A,B)−s(Y;A,B) or the difference s(A,B;X,Y)=s(A;X,Y)−s(B;X,Y), while the effect sizes differ, which is another reason why we favored nonstandardized measures in our analysis.

### Match between corpora and levels of individualism and economic development

Finally, to analyze how GS(A,B) vary with country level of individualism and economic development, we relied on available data from Wikipedia outlining the relative contribution of each country to each corpus (Wikimedia Foundation). In our main specification, we essentially considered for each text corpus the level of individualism or of economic development of the country that contributes the most to the corpus (see details and specific examples in the Supplementary material). In robustness checks, we considered alternative specifications and in particular a specification where we adopt for each corpus the weighted average of the levels of individualism and development of the countries contributing to the corpus, based on countries’ relative contribution to the considered corpus, as in (17, 20) and a specification where we limit the sample of corpora to those for which one country contributes to more than 50%. Table S10 shows that our results are robust to these various matches between corpora and macro variables.

## Supplementary Material

pgad355_Supplementary_Data

## Data Availability

We used pretrained word vectors, which were generated by training on Wikipedia and Common Crawl datasets using fastText (34, 35). These word vectors are publicly accessible and can be found at the following links: https://fasttext.cc/docs/en/crawl-vectors.html and https://fasttext.cc/docs/en/pretrained-vectors.html. All the codes used for our main analyses are publicly available via the Open Science Framework and can be accessed at https://osf.io/gkmv4/.

## Footnotes

1
https://stats.wikimedia.org/wikimedia/animations/wivivi/wivivi.html.
