# Supplementary material for: Gender stereotypes embedded in natural language are stronger in more economically developed and individualistic countries
Source: PNAS Nexus. 2023 Nov 21;2(11):pgad355. doi: 10.1093/pnasnexus/pgad355 (PMC10662454; doi:10.1093/pnasnexus/pgad355)
Supplement: pgad355_Supplementary_Data [file pgad355_supplementary_data.docx]

**
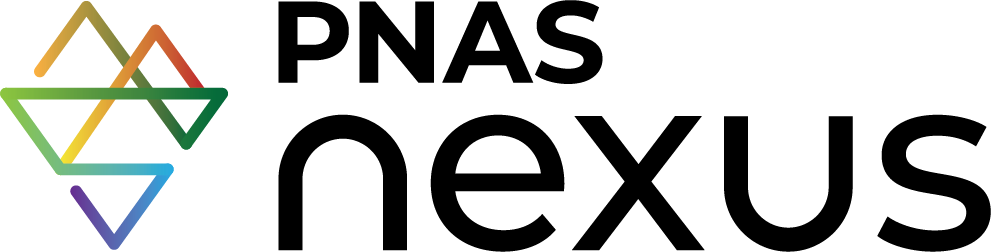
**

**Supplementary Information for**

Gender stereotypes embedded in natural language are stronger in more economically developed and individualistic countries

**Author:** Clotilde Napp^a,b,*^

**Affiliations:**

^a^ CNRS, UMR7088, 75016 Paris, France

^b^ Université Paris-Dauphine, PSL Research University, Paris, France

^*^Corresponding author. **Email:**  [clotilde.napp@dauphine.psl.eu](mailto:xxxxx@xxxx.xxx)

**This PDF file includes:**

Supplementary text: Appendix A, B

Figures S1 to S5

Tables S1 to S11

SI References

**Structure of the SI**

[Supplementary Information Text 2](#_Toc142841810)

[Appendix A: Methods 2](#_Toc142841811)

[Word embeddings 2](#_Toc142841812)

[Text corpora and algorithm 2](#_Toc142841813)

[Word Embedding Association Test (WEAT) 2](#_Toc142841814)

[Measures GS of Gender Stereotypes 3](#_Toc142841815)

[Stimuli 3](#_Toc142841816)

[Translations 4](#_Toc142841817)

[Single Category WEAT 4](#_Toc142841818)

[Match between corpora and levels of individualism and economic development 5](#_Toc142841819)

[Appendix B: Data 5](#_Toc142841820)

[Data on country-level measures of economic development and wealth 5](#_Toc142841821)

[Data on country-level measures of individualism 6](#_Toc142841822)

[Data on country-level measures of gender equality 6](#_Toc142841823)

[Data on country-level measures of ecological stress 7](#_Toc142841824)

[Other Data 7](#_Toc142841825)

[Appendix C: Figures and Tables 8](#_Toc142841826)

**Supplementary Information Text**

Appendix A: Methods

## Word embeddings

To identify gender stereotypes embedded in text corpora, we used *word embeddings* to quantify the association between groups (male-female) and attributes (*e.g.*, career-family, (1)). The idea underlying word embeddings is that patterns of word co-occurrences are modelled (with machine-learning algorithms) to quantify the semantic relationships between words (e.g., the semantic relationship between the words *women* and *family* vs. *men* and *family*).

Word embeddings derive their power from the discovery that vector spaces with around 300 dimensions suffice to capture most aspects of similarity, enabling a computationally tractable representation of all or most words in large corpora of text (2). Representing words as vectors (*i.e.*, as word embeddings) permits to quantify their semantic similarity.

As explained in the Materials and Methods section of the main text, the level of similarity between two words $w_{1}$ and $w_{2}$ with corresponding word embeddings $\vec{w_{1}}$ and $\vec{w_{2}}$ is defined as the cosine similarity $\cos\left( \vec{w_{1}},\vec{w_{2}} \right)=\vec{w_{1}}{\cdot\vec{w_{2}}}/\parallel\vec{w_{1}}\parallel\parallel\vec{w_{2}}\parallel$, where the numerator represents the inner product of the embeddings $\vec{w_{1}}$ and $\vec{w_{2}}$, while the denominator is the product of their respective Euclidean norms.

Word embeddings can be used to identify the strength of gender stereotypes within a given corpus. If a corpus documents a gender stereotype, for instance associating men more than women with career versus family, then words representing men compared to those representing women will appear semantically more related with career words versus family words. The method has already shown feasibility in documenting social group representations, including gender biases (e.g., (1, 3–5)).

## Text corpora and algorithm

We used two pretrained publicly available word embeddings, the Wikipedia embedding and the Common Crawl embedding. These embeddings were respectively trained on text data from Wikipedia and from the Common Crawl project across more than 100 languages and produced by (6) and (7) respectively. For both datasets, each embedding consists of a vector of 300 dimensions for each word, derived from one of the latest and most widely used algorithm, namely the fastText algorithm (a variant of word2vec). Wikipedia provides information about the relative contribution of each language corpus to the whole Wikipedia and about the relative contribution of each country to each language corpus (Wikimedia Foundation^[[1]](#footnote-1)^). This information is not available for all language corpora, and we kept in our sample those for which we had this information. Moreover, we excluded from our analysis languages that are not an official language in any country^[[2]](#footnote-2)^. There are several official languages in India, and we kept Hindi and grouped together the six most important languages after Hindi (Tamil, Marathi, Malayalam, Telugu, Kannada, Gujarati) into a single observation (denoted by R_India). Our final sample consisted of 82 language corpora from Wikipedia and 75 language corpora from Common Crawl. In robustness checks (Table S10), we considered a restriction of the sample to the largest corpora in Wikipedia using the same information from the Wikimedia Foundation. More precisely, we restricted the sample to the corpora contributing to more than 0.01% of the total worldwide Wikipedia, which essentially consists of the 50 most important contributors in our sample.

## Word Embedding Association Test (WEAT)

To transform the individual word-embedding vectors into measures of gender stereotypes, we used the Word Embedding Association Test (WEAT), a method introduced by (1) and widely applied (*e.g.*, (3, 8)).

The WEAT computes a measure of the relative association between words representing group categories (in our case, male-female) and words representing attributes (for instance career-family). The degree of association is measured from the cosine similarities between category and attribute word-embedding vectors.

Borrowing terminology from the Implicit Association Test (IAT, (9, 10)), the Word Embedding Association Test (WEAT) measures the association between two sets of target words $X$ and $Y$ (*e.g.*, a set of male words and a set of female words), and two sets of attribute words $A$ and $B$ (*e.g.*, a set of career words and a set of family words). The WEAT tests the null hypothesis that there is no difference between the two sets of target words in terms of their relative similarity to the two sets of attribute words.

More precisely, the level of similarity between two words $w_{1}$ and $w_{2}$ is defined as above by the cosine similarity between their embeddings $\vec{w_{1}}$ and $\vec{w_{2}}$. As explained in the Materials and Methods section, the relative level of similarity of a target word $w$ to the set of words $A$ versus the set $B$ is given by $s\left( w,A,B \right)={mean}_{a\in A}\cos\left( \vec{w},\vec{a} \right)-{mean}_{b\in B}\cos\left( \vec{w},\vec{b} \right)$. The differential association of the two sets of target words $\left( X,Y \right)$ with the attributes $\left( A,B \right)$ is given by $s\left( X,Y;A,B \right)={mean}_{x\in X}s\left( x,A,B \right)-{mean}_{y\in Y}s\left( y,A,B \right)$. The normalized differential association or effect size is given by $ES\left( X,Y;A,B \right)={s\left( X,Y;A,B \right)}/{{std dev}_{w\in X\cup Y}s\left( w,A,B \right)}$.

## Measures GS of Gender Stereotypes

We focused on gender stereotypes that have been robustly documented. Specifically, we examined the male-career/female-family association, the male-math/female-liberal arts association and the male-science/female-arts association.

Applying the WEAT permits to obtain measures of these gender stereotypes for each corpus. We considered as sets of target words a set $X$ of male words, and a set $Y$ of female words. The sets of attribute words change with the gender stereotype under consideration, with a set $A$ of career words (resp. math, science words) and a set $B$ of family words (resp. liberal arts, arts words) for gender career-family (resp. math-liberal arts, science-arts) stereotypes. For each stereotype represented by the sets $\left( A,B \right)$, we took as our measure of gender stereotypes $GS\left( A,B \right)\equiv s\left( X,Y;A,B \right)$. Higher values of $GS(A,B)$ correspond to stronger gender stereotypes $\left( A,B \right)$ embedded in the text corpus.

Note that unlike the normalized measure $ES(A,B)$, the measure $GS(A,B)$ is linear and permits to easily collapse gender stereotypes or data sources since $GS\left( A\cup A',B\cup B' \right)=[GS\left( A,B \right)+GS\left( A',B' \right)]/2$.

Since the gender associations related to math and to science are related, we collapsed them to obtain a measure of the stereotyped association male-math or science/ female-liberal arts or arts. More precisely, for $(A,B)$= (math, liberal arts) and $(A',B')$= (science, arts), gender stereotypes about math/science-liberal arts/arts are given by $GS\left( A\cup A',B\cup B' \right)=[GS\left( A,B \right)+GS\left( A',B' \right)]/2$.

We also considered for each corpus a measure of aggregate gender stereotypes by considering a weighted average of the (non-standardized) measures of gender stereotypes. More precisely, for $(A,B)$= (career, family), $(A',B')$= (math, liberal arts) and $(A'',B'')$ = (science, arts), the measure of aggregate stereotype is given by $GS\left( aggregate \right)=[GS\left( A,B \right)+GS\left( A'\cup A'',B'\cup B'' \right)]/2$.

Finally, we defined the measure of gender stereotype $GS$ on both sources Wikipedia and Common Crawl by the average of the measure of gender stereotype $GS$ on the Wikipedia corpus and the measure of gender stereotype $GS$ on the Common Crawl corpus.

## Stimuli

To select word stimuli (in English), we began with the stimuli lists from (1), that used the same stimuli as in the Implicit Association Tests.

Our linguistic stimuli were a set of female and a set of male ‘target words’ that remain the same for the analysis of all gender stereotypes and two sets of ‘attribute words’ used in the Project Implicit, that change with the stereotype under consideration.

**Male-female stimuli**

The word stimuli to represent women and men categories are the same eight words as in (10) and (1):

• Male words:

$A$ = ["male", "man", "boy", "brother", "he", "him", "his", "son"]

• Female words:

$B$ = ["female", "woman", "girl", "sister", "she", "her", "hers", "daughter"]

Note that we do not consider proper names because they are not easy to translate.

**Career-family stimuli**

The word stimuli to represent career and family attributes are the same as in (10) and (1):

• Career words:

$X$ = ["executive", "management", "professional", "corporation", "salary", "office", "business", "career"]

• Family words:

$Y$ = ["home", "parents", "children", "family", "cousins", "marriage", "wedding", "relatives"]

**Math-liberal arts stimuli**

The word stimuli to represent math and liberal arts attributes are the same as in (10) and (1):

• Math words:

$X$ = ["math", "algebra", "geometry", "calculus", "equations", "computation", "numbers", "addition"]

• Arts Words:

$Y$ = ["poetry", "art", "dance", "literature", "novel", "symphony", "drama", "sculpture"]

**Science-arts stimuli**

The word stimuli to represent science and arts attributes are the same as in (11) and (1):

• Science words:

$X$ = ["science", "technology", "physics", "chemistry", "Einstein", "NASA", "experiment", "astronomy"]

• Arts words:

$Y$ = ["poetry", "art", "Shakespeare", "dance", "literature", "novel", "symphony", "drama"]

In robustness checks, we considered alternative sets of stimuli, as in (12). We kept the same sets of eight male and female words, but considered the following stimuli for the analysis of career-family stereotypes and science-arts stereotypes:

**Alternative Career-family stimuli**

$X$ = ["work", "office", "job", "business", "trade", "activity", "act", "money"]

$Y$ = ["baby", "house", "home", "wedding", "kid", "family", "marry", "children"]

**Alternative Science-arts stimuli**

$X$ = ["science", "scientist", "physic", "engineer", "space", "spaceship", "astronaut", "microscope"]

$Y$ = ["art", "dance", "sing", "paint", "painting", "song", "draw", "drawing"]

## Translations

Each text corpus relies on a different language, and to calculate a measure of biased associations from word embedding models trained on text in other corpora than the one in English, we translated all the English stimuli using Google Translate. While the coverage of Google Translate service is significant, it is not comprehensive and 5 languages were not covered by Google Translate (Egyptian Arabic, Serbo-croatian, Cantonese, Nynorsk, Haitian). For them we used ChatGPT to obtain translations.

Moreover, we considered the robustness of our results to another translation tool. We considered translations provided by ChatGPT for all languages. More precisely, for each language we asked ChatGPT to provide "the most common translations" for the sets of target words. Table S11 shows that the relations between gender stereotypes GS and measures of individualism and GNI are robust to the change of translation tool.

## Single Category WEAT

A limitation of the WEAT, as well as the traditional IAT, is that it condensates into one measure the relative associations of attribute sets against two target sets, and loses the similarity against one single target set. The single category WEAT permits to disentangle the two relative associations, by measuring the association between one set of target words $W$ and two sets of attribute words $A$ and $B$.

With the same notations as in the Materials and Methods section, the relative similarity of the target set $X$ to the attribute set $A$ versus $B$ is then given by $s\left( X;A,B \right)={mean}_{x\in X}s\left( x,A,B \right)$ and the relative similarity of the target set $Y$ to the attribute set $A$ versus $B$ by $s\left( Y;A,B \right)={mean}_{y\in Y}s\left( y,A,B \right)$.

Analogously, the relative similarity of the attribute set $A$ to the set $X$ versus $Y$ is given by $s\left( A;X,Y \right)={mean}_{a\in A}s\left( a,X,Y \right)$ and the relative similarity of the attribute set $B$ to the set $X$ versus $Y$ by $s\left( B;X,Y \right)={mean}_{b\in B}s\left( b,X,Y \right)$.

In the case of the gender career-family stereotype, then $s\left( X;A,B \right)$ represents the extent to which male words are more similar to career words than family words, (the opposite of) $s\left( Y;A,B \right)$ the extent to which female words are more similar to family words than career words, $s\left( A;X,Y \right)$the extent to which career words are more masculine than feminine and (the opposite of) $s\left( B;X,Y \right)$ the extent to which family words are more feminine than masculine.

For all stereotypes $\left( A,B \right)$, our measure of gender stereotype $GS\left( A,B \right)$ is indifferently given by $s\left( X,Y;A,B \right)=s\left( X;A,B \right)-s\left( Y;A,B \right)$ or by $s\left( A,B;X,Y \right)=s\left( A;X,Y \right)-s\left( B;X,Y \right)$, while the effect sizes differ.

## Match between corpora and levels of individualism and economic development

While measures of individualism (such as Hofstede's measure of individualism) and economic development (such as the Gross National Income) are available by country, measures of gender stereotypes are available by text corpora, and each text corpus corresponds to a unique language but not necessarily to a unique country. To associate a level of individualism and of economic development to each language corpus, we utilized data from Wikipedia outlining the relative contribution of each country to each language project (Wikimedia Foundation^[[3]](#footnote-3)^), as in (3). There are corpora for which one country is nearly the only contributor, in which case it is natural to consider the level of individualism/GNI of this country. For instance, for the Japanese corpus, the relative contribution of each country is the following: Japan 96.5%, and some minor contributors (e.g., US 1%, Taiwan 0.4%, South Korea 0.2%, Hong Kong 0.2%). However, for other corpora, the situation is less obvious, several countries being large contributors. For instance, for the English corpus, the relative contribution of each country is the following: US 42%, UK 10%, India 8%, Canada 3%, Australia 3% and some more minor contributors. For the Spanish corpus, the relative contribution is the following: Mexico 22%, Spain 20%, Argentina 12%, Colombia 10%, Chile 6% and some more minor contributors.

In our main setting, we associate with each language corpus the level of individualism and of gross national income of the country that contributes the most to the language project (only considering countries having the language of the corpus as official language^[[4]](#footnote-4)^). For instance, the level of individualism of the Japanese corpus is given by Japan's level of individualism. The level of individualism of the English corpus is given by US level of individualism and the level of individualism of the Spanish corpus is given by Mexico's level of individualism.

In robustness checks (Table S10), we consider the following alternative matches between corpora and levels of individualism/GNI. First, we consider *absolute* contribution rather than *relative* contribution: we first restrict the sample to corpora to which one country contributes to more than 50%, and then take for each corpus the level of individualism and of GNI of the given country. Under such a specification, we keep for example the Japanese corpus (and take Japan's level of individualism as its level of individualism) but exclude the English and the Spanish corpora. Second, we keep all corpora but take as the level of individualism and of economic development of a given corpus the weighted average of the levels of individualism and development of the countries contributing to the corpus, based on countries' relative contribution to the considered corpus (only considering countries contributing to more than 10% of the corpus and having the language of the corpus as official language). A similar method is applied in (3). In this specification, the level of individualism of the English corpus is given by $Ind\left( English \right)=[0.42 Ind(US)+0.1 Ind(UK)]/0.52$, where for each country $C$, $Ind\left( C \right)$ denotes its level of individualism, and the level of individualism of the Spanish corpus is given by $Ind\left( Spanish \right)=[0.22 Ind(Mexico)+0.2 Ind(Spain)+ 0.12 Ind(Arg.)+0.1 Ind(Col.)]/0.64$. Third, we adopt the 'naive' match of attributing to each language corpus the level of individualism of the country with most speakers of the considered language.

Appendix B: Data

## Data on country-level measures of economic development and wealth

*Gross National Income (GNI)*

GNI per capita is the dollar value of a country's final income in a year, divided by its midyear population. Our main measure of GNI is the value provided by the World Bank, using the Atlas method, for years 2020-2022. The Atlas method smoothes exchange rate fluctuations by using a three-year moving average, price-adjusted conversion factor.

*Source:* GNI (nominal) per capita 2020, World Bank

*https://en.wikipedia.org/wiki/List_of_countries_by_GNI_(nominal)_per_capita*

*Gross Domestic Product (GDP)*

GDP per capita is gross domestic product divided by midyear population. GDP is the sum of gross value added by all resident producers in the economy plus any product taxes and minus any subsidies not included in the value of the products. It is calculated without making deductions for depreciation of fabricated assets or for depletion and degradation of natural resources.

Our main measure of GDP is the average of the values for 2005, 2010, 2015 and 2020. Data are in constant 2010 U.S. dollars.

We also consider historical values of GDP per capita: GDP 1960, GDP 1970, GDP 1980, GDP 1990 (in Table S6B). Data are in constant 2015 U.S. dollars.

*Source:*

Main value: GDP PPP cstt 2010, World Bank

*https://data.worldbank.org/indicator/NY.GDP.PCAP.PP.KD?end=2019&most_recent_year_desc=true&start=2018*

Historical values: GDP1960-1970-1980-1990 PPP cstt 2015, World Bank

*https://data.worldbank.org/indicator/NY.GDP.PCAP.KD*

*Human Development Index (HDI)*

The Human Development Index (HDI) is a composite statistic of life expectancy, education, and per capita income indicators. A country scores a higher HDI when life expectancy, education level and per capita income are higher. Our main value of HDI is the average of the values in 2000, 2010, 2015 and 2019.

*Source:* HDI2019, Human development report

*https://hdr.undp.org/en/content/human-development-index-hdi*

## Data on country-level measures of individualism

*Individualism*

Our main variable of individualism is based on Hofstede’s cultural dimensions (13). It captures the degree to which a society is individualistic as opposed to collectivist, *i.e.,* the extent to which individuals are integrated into groups, and how loose are social links. Individualism stands for a society in which the ties between individuals are loose, where individuals are expected to look after their immediate family only. Its opposite, collectivism, stands for a society in which individuals from birth onwards are integrated into strong, cohesive in-groups, which look after them in exchange for unquestioning loyalty. A society’s position on this dimension is reflected in whether people’s self-image is defined in terms of “I” or “we.”

*Source:* *https://www.hofstede-insights.com/product/compare-countries/*

*In-group Collectivism*

We consider as another indicator of individualism the opposite of the indicator of In-group collectivism (Societal Practices) from the Globe survey about cultural practices and values (14). It captures the degree to which individuals express pride, loyalty, and cohesiveness in their families. It relies on the extent to which respondents agree on a seven-point scale with the four following items:

- In this society, children take pride in the individual accomplishments of their parents
- In this society, parents take pride in the individual accomplishments of their children
- In this society, aging parents generally live at home with their children
- In this society, children generally live at home with their parents until they get married

See https://globeproject.com/data/GLOBE-Dimensions-Definitions-and-Scale-Items.pdf for more details on the definition. We consider the opposite of this indicator, denoted by M-Collectivism, so that larger values correspond to more individualism.

*Source*: Globe Project

https://globeproject.com/study_2004_2007#data

## Data on country-level measures of gender equality

*Equality values*

The equality values (sub-)index is provided by the World Value Surveys. It encompasses questions about equality between men and women in education, in politics, and in the labor force and more precisely, about the three following items:

- A university education is more important for a boy than for a girl (Education)
- On the whole, men make better political leaders than women do (Politics)
- When jobs are scarce, men should have more right to a job than women (Jobs)

Larger values correspond to respondents having more egalitarian views regarding the roles of women and men.

*Source*: World Value Survey, Wave 7 (2017-2020)

*https://www.worldvaluessurvey.org/WVSOnline.jsp*

*Gender Gap Index (GGI)*

The Gender Gap Index, from the World Economic Forum, synthesizes the position of women in any given country by taking into account economic opportunities, economic participation, educational attainment, political achievements, and health and well-being. Larger values point to a better position of women in society.

*Source:* GGI2018, World economic forum

*http://www3.weforum.org/docs/WEF_GGGR_2018.pdf*

*Female Labor Force Participation*

Participation of women in the labor force is part of gender equality (in practice). To measure it, we consider the ratio of female to male labor force participation rates. We consider the mean of these ratios between 2010 and 2020.

*Source:World Bank*

https://data.worldbank.org/indicator/SL.TLF.CACT.FM.ZS

*Women's fertility rate*

Women's fertility rate is also part of gender equality (in practice) and can impact gendered beliefs about career and family. Data are from 2019.

*Source:* Fertility rate, total (births per woman) *World Bank*

## Data on country-level measures of ecological stress

The two most prominent sources of ecological stress are disease and lack of nutrition. We consider the same measures and data for ecological stress as in (15), using both historical and contemporary estimates.

*Nutrition*

As an indicator of nutrition availability, the per-capita calorie consumption for the countries included in the study Food and Agriculture Organization of the United Nations was used. This data represents the estimated amount of food energy available for human consumption, measured in calories, based on each country's domestic food supply. Historical and contemporary per capita calorie consumption respectively correspond to the average estimate for the years 1961 to 1973 and for the years 2001 to 2011.

*Source*: (15)

*Disease*

As an indicator of disease prevalence, data provided by (16) were used. Historical pathogen prevalence was estimated using historical infectious disease atlases published between 1944 and 1961, along with other historical information. Contemporary prevalence data was obtained from the GIDEON database on infectious diseases. The measurement consists of prevalence scores for seven classes of pathogens (leishmanias, trypanosomes, malaria, schistosomes, filariae, spirochetes, and leprosy), which were summed to create a composite score representing global pathogen stress.

*Source*: (15)

## Other Data

*Language Genderedness*

World languages can be categorized as gendered (a language in which the form of noun, verb, or pronoun is presented as female or male) versus genderless languages. We relied on data from (3), that provides a division of the 45 languages they consider based on whether they are gendered or genderless.

*Source*:(3)

*Language Family*

Languages can belong to different language families (*e.g.*, Indo-European, Turkic, Sino-Tibetan). We proceeded as in (3) to classify the languages in our sample using data from Ethnologue.

*Source*: Simmons, G. F., & Fennig, C. D. (2018). Ethnologue: Languages of the World. Retrieved from https://www.ethnologue.com/

Appendix C: Figures and Tables

0

5

10

15

Density

-.05

0

.05

.1

Stereotypes about career and family

0

5

10

15

20

Density

-.05

0

.05

.1

Stereotypes about math and liberal arts

0

5

10

15

20

Density

-.05

0

.05

.1

Stereotypes about science and arts

**Figure S1: Histograms of linguistic gender stereotypes. Wikipedia Corpus.**

The figure presents the histograms of measures of language gender stereotypes about career-family, math-liberal arts, science-arts relying on text corpora in 82 different languages from Wikipedia. Details about data and methods are provided in Appendix A.


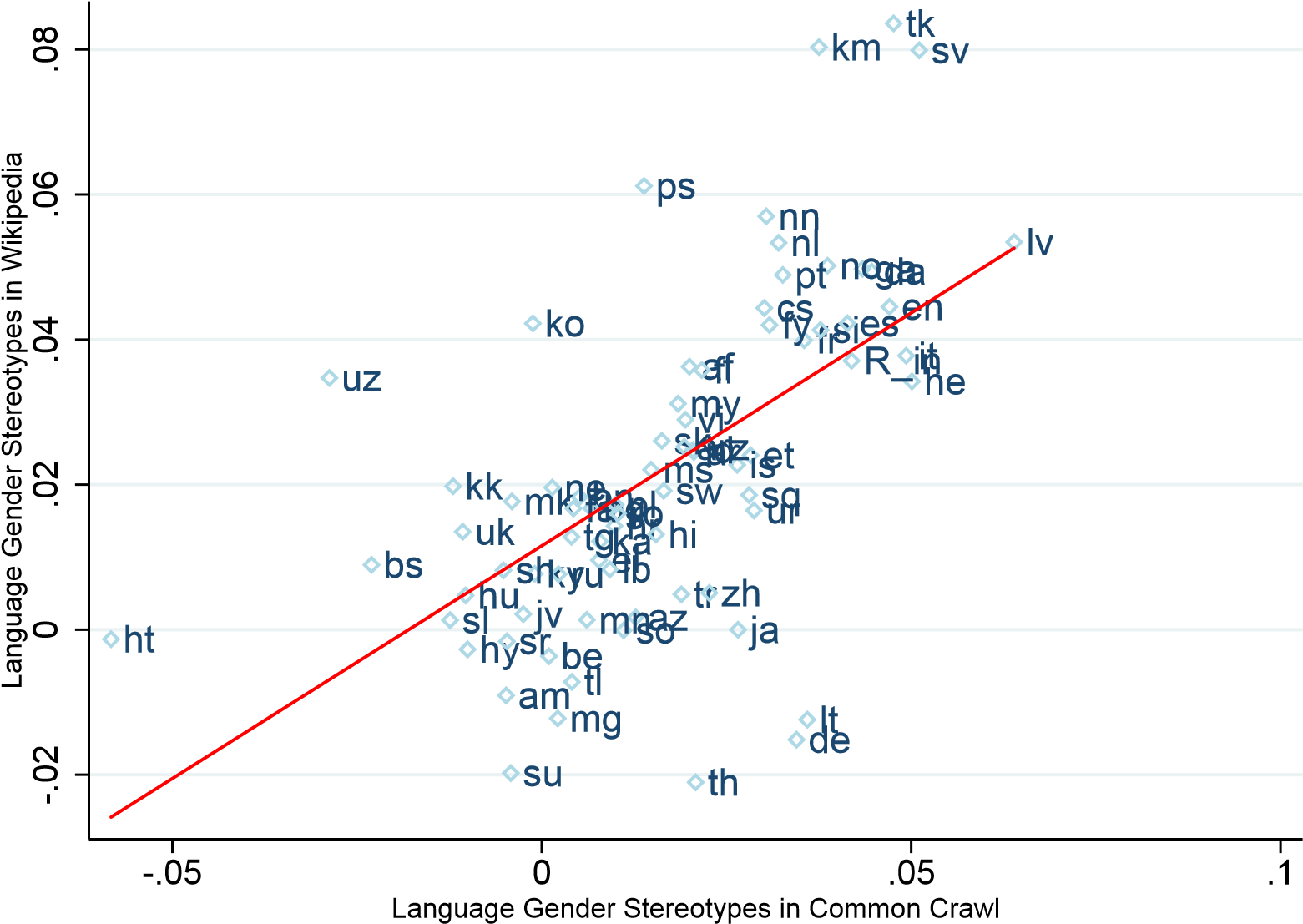


**Figure S2: Relation between linguistic gender stereotypes measured on Wikipedia and on Common Crawl Corpora**

The figure presents the relation between the measure of language aggregate gender stereotype (about career, math and science) relying on Wikipedia corpora and the measure of language aggregate gender stereotype relying on Common Crawl corpora. The figure is based on 75 observations (language corpora). See Appendix A for details about the measure of language aggregate gender stereotypes. Language codes from ISO 639.1.


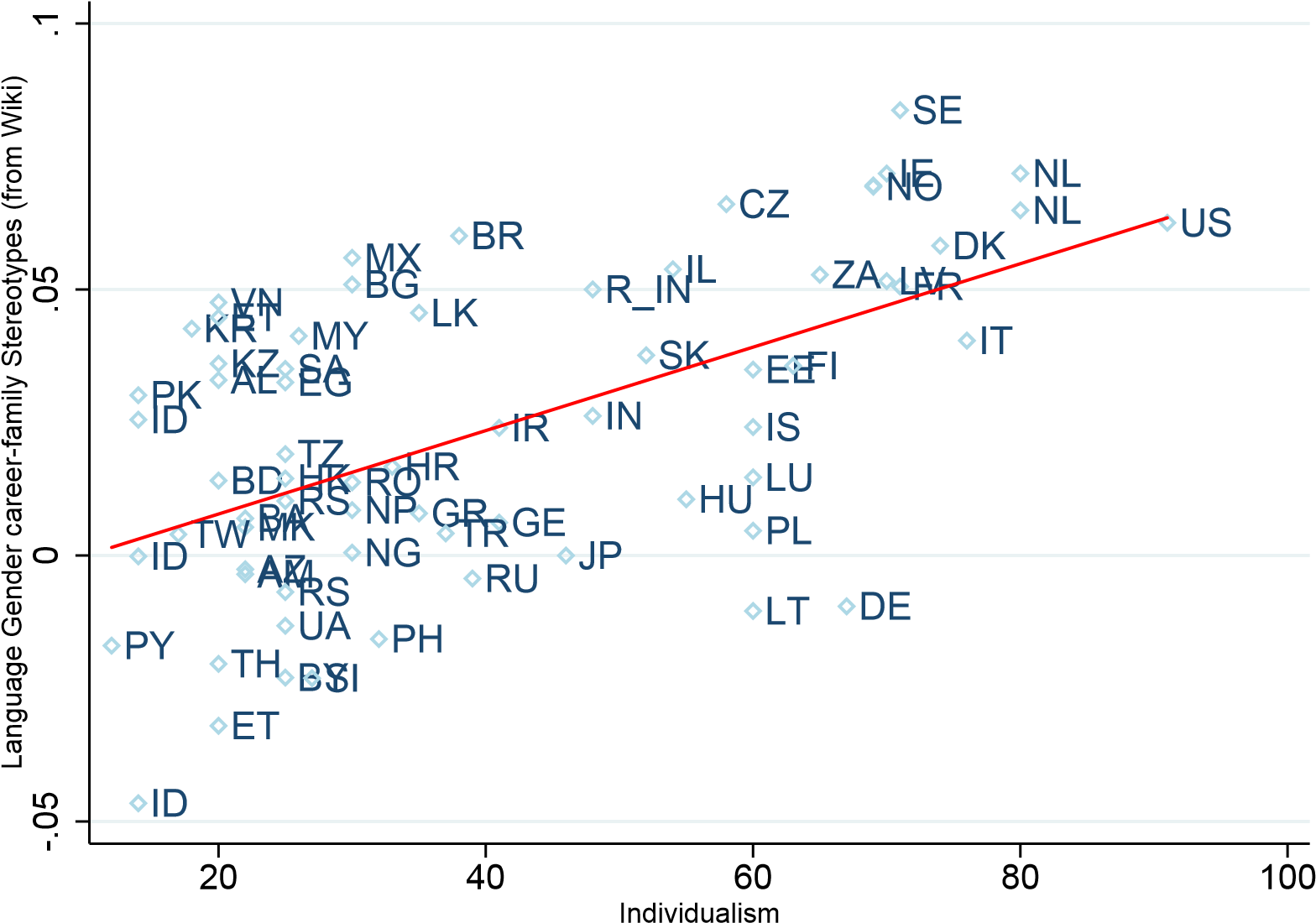


ZA

AL

ET

SA

AM

AZ

BY

BD

BA

BG

HR

CZ

DK

NL

US

EE

I

~~F~~

FR

GE

DE

GR

IN

HU

IS

ID

IE

IT

JP

KZ

KR

LV

LT

LU

MK

MY

NP

O

N

IR

PL

BR

RO

U

~~R~~

RS

LK

SK

SI

MX

TH

VN

UA

IL

SE

PH

TR

PK

TW

TZ

ID

EG

RS

NO

ID

NL

R_IN

-.05

0

.05

.1

Language Gender career-family Stereotypes (from Common Crawl)

20

40

60

80

100

Individualism


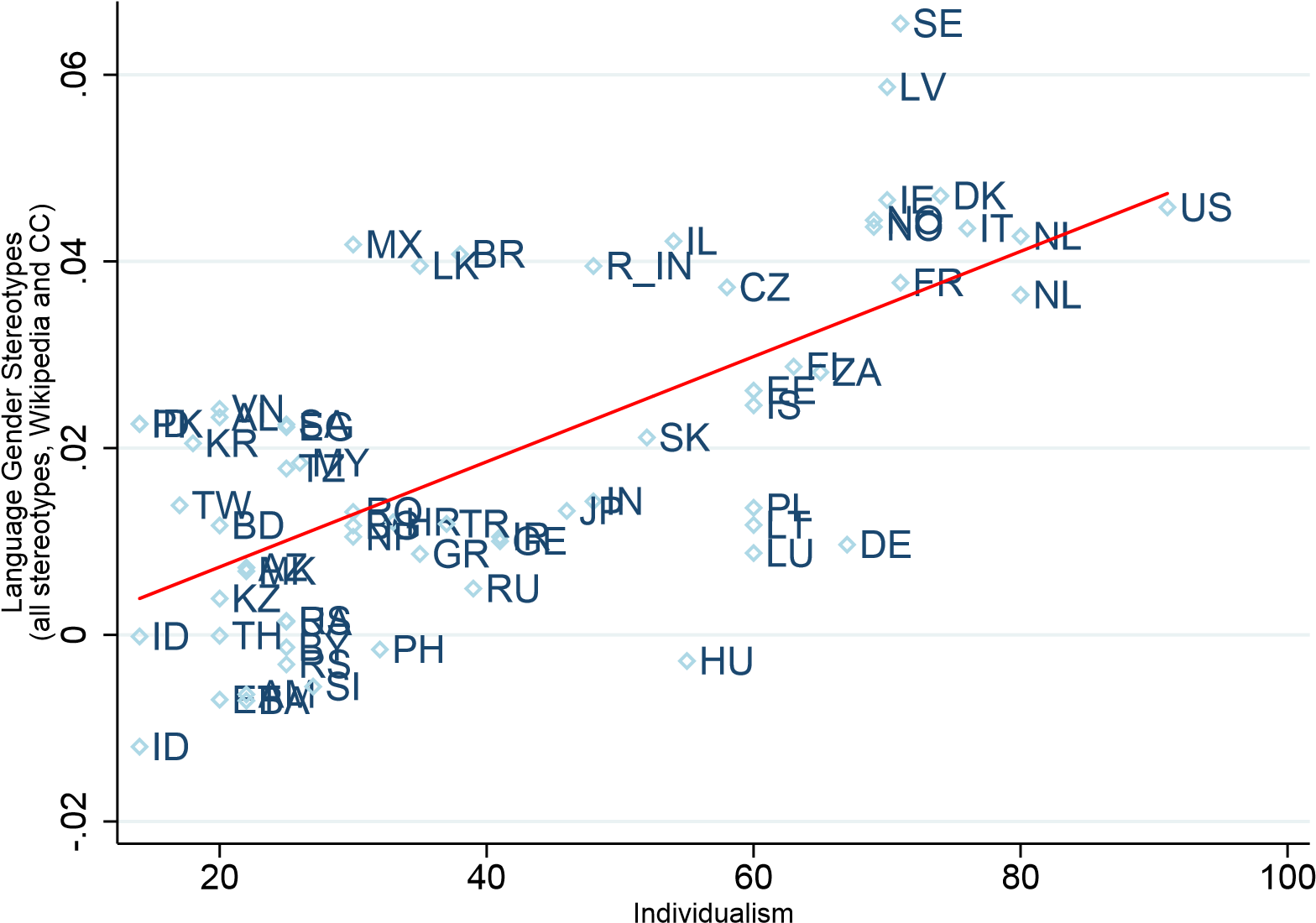


**Figure S3: Relation between linguistic gender stereotypes and country levels of individualism.**

The Figure presents measures of language gender stereotypes as a function of country level of individualism (Hofstede's measure of individualism). The top figures consider gender stereotypes about career and family embedded in the text corpora of the Wikipedia (first figure) and Common Crawl (second figure) projects, while the third figure considers the three gender stereotypes about career, math and science embedded in text corpora from both sources, Wikipedia and Common Crawl. Gender stereotypes are measured through the Word Embedding Association Test (1). Details about methods and data are provided in Appendix A. Country codes from ISO 3166.


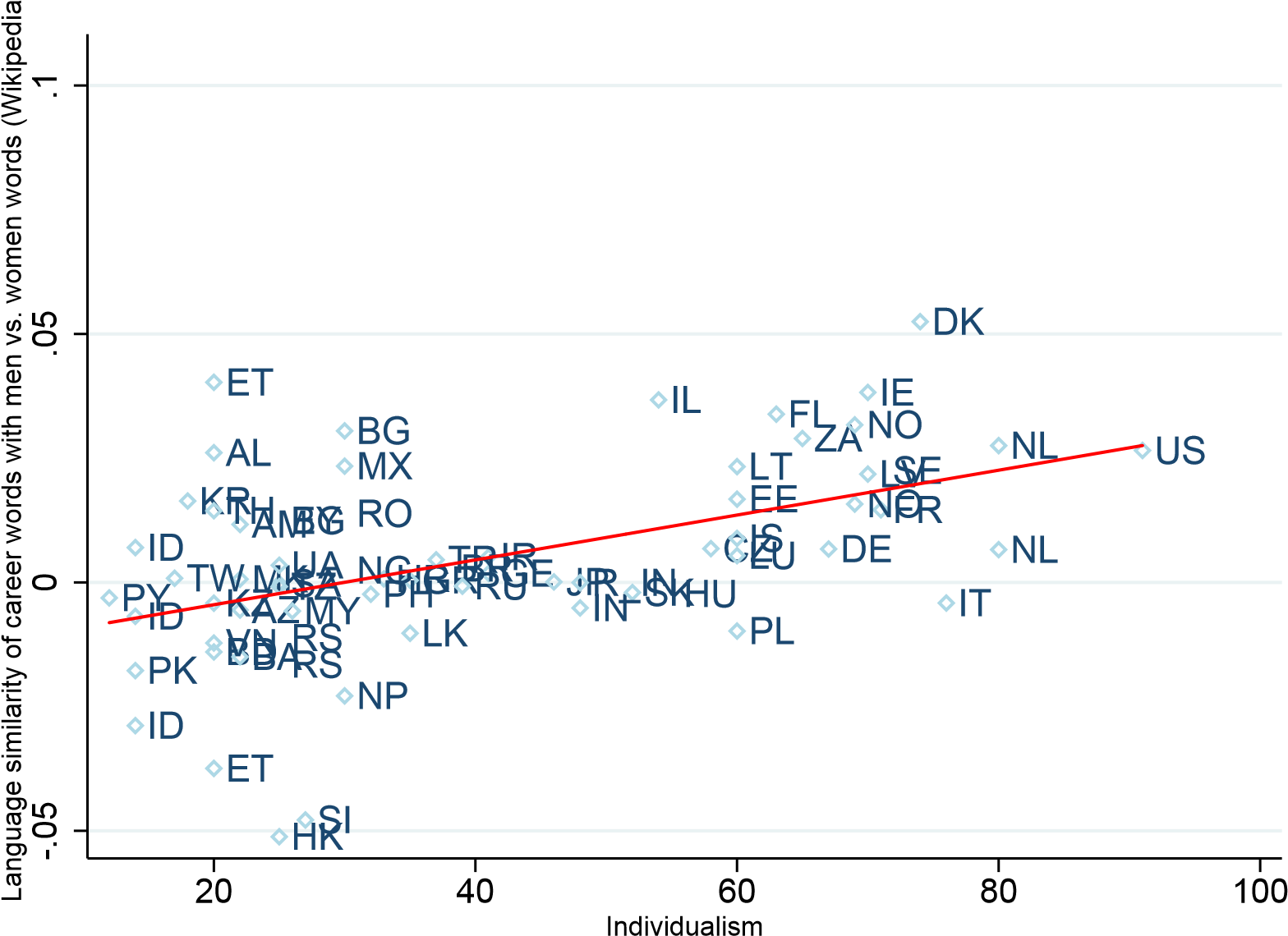


**Figure S4A: Similarity of career words with male vs. female words as a function of country individualism. Wikipedia source**

The figure presents the relative association of career words with male vs. female words, relying on single-category WEAT, as a function of country level of individualism (Hofstede's measure of individualism). Details about the measure of association and about data sources are provided in Appendix A and B. Country codes from ISO 3166.


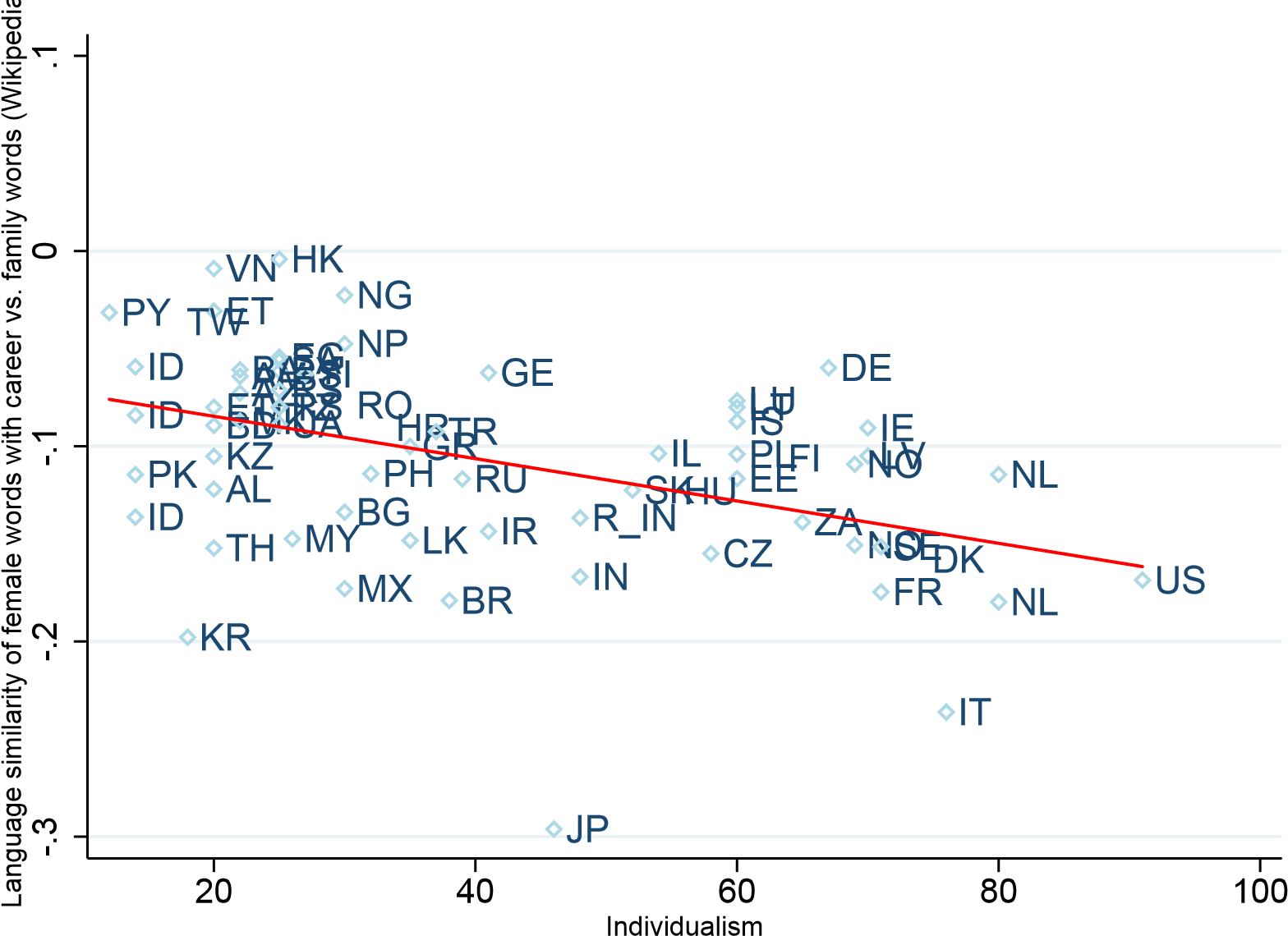


**Figure S4B: Similarity of female words with career vs. family words as a function of country individualism. Wikipedia source**

The figure presents the relative association of female words with career vs. family words, relying on single-category Word Embedding Association Test, as a function of country level of individualism (Hofstede's measure of individualism). Details about the measure of association and about data sources are provided in Appendix A and B. Country codes from ISO 3166.


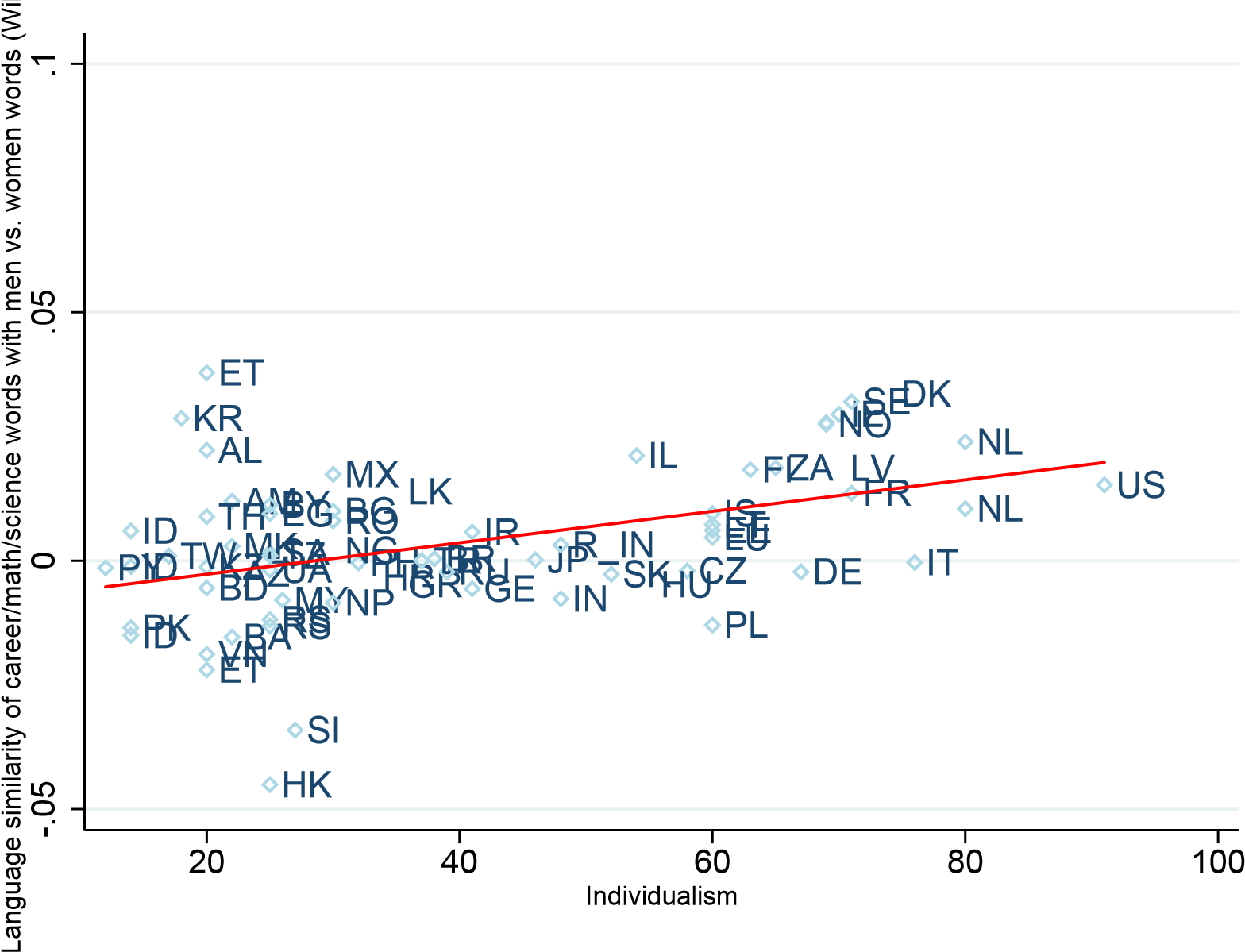


**Figure S5: Similarity of career/math/science words with male vs. female words as a function of country individualism. Wikipedia source**

The figure presents the relative association of career/math/science words with male vs. female words, relying on single-category Word Embedding Association Test, as a function of country level of individualism (Hofstede's measure of individualism). Details about the measure of association and about data sources are provided in Appendix A and B. Country codes from ISO 3166.

**Table S1: Linguistic gender stereotypes about career-family, math-liberal arts, science-arts by language corpus. Wikipedia and Common Crawl sources**

|  | **Career-Family** | | | **Math-Liberal Arts** | | | | | **Science-Arts** | | |  |
| --- | --- | --- | --- | --- | --- | --- | --- | --- | --- | --- | --- | --- |
|  | Wikipedia | Common Crawl | | | Wikipedia | | Common Crawl | | | Wikipedia | Common Crawl |  |
| All Language Corpora | |  | | |  | |  | | |  |  |  |
| Mean | 0,0240  (0.0034) | 0,0175  (0.0037) | | | 0,0216  (0.0024) | | 0,0201  (0.0026) | | | 0,0151  (0.0024) | 0,0087  (0.0024) |  |
| Std Dev. | 0.0305 | 0.0319 | | | 0.0220 | | 0.0224 | | | 0.0215 | 0.0206 |  |
|  |  |  | | |  | |  | | |  |  |  |
| OBS. | 82 | 75 | | | 82 | | 75 | | | 82 | 75 |  |
| Min. | -0.0466 | -0.0820 | | | -0.0300 | | -0.0363 | | | -0.0373 | -0.0463 |  |
| Max. | 0.0979 | 0.0873 | | | 0.0863 | | 0.0692 | | | 0.1027 | 0.0647 |  |
|  |  |  | | |  |  | | |  | |  |  |
| By Language Corpus | |  | |  | |  | | |  | |  |  |
| Afrikaans | 0,053 | | 0,028 | | 0,020 | | | 0,009 | | 0,019 | 0,016 | |
| Albanian | 0,033 | | 0,036 | | 0,010 | | | 0,030 | | -0,002 | 0,010 | |
| Amharic | -0,032 | | -0,017 | | 0,013 | | | 0,013 | | 0,014 | 0,001 | |
| Arabic | 0,035 | | 0,031 | | 0,014 | | | 0,010 | | 0,013 | 0,009 | |
| Armenian | -0,004 | | -0,019 | | 0,001 | | | 0,005 | | -0,005 | -0,008 | |
| Azerbaijani | -0,003 | | 0,006 | | 0,007 | | | 0,026 | | 0,005 | 0,013 | |
| Belarusian | -0,023 | | -0,015 | | 0,027 | | | 0,021 | | 0,004 | 0,012 | |
| Bengali | 0,014 | | -0,003 | | 0,027 | | | 0,018 | | 0,018 | 0,008 | |
| Bosnian | 0,007 | | -0,035 | | 0,029 | | | 0,000 | | -0,008 | -0,022 | |
| Bulgarian | 0,051 | | 0,015 | | -0,020 | | | 0,004 | | -0,015 | -0,007 | |
| Burmese | 0,039 | | 0,028 | | 0,027 | | | 0,015 | | 0,019 | 0,003 | |
| Cantonese | 0,014 | |  | | 0,020 | | |  | | -0,037 |  | |
| Chinese | 0,004 | | 0,000 | | 0,007 | | | 0,047 | | 0,005 | 0,045 | |
| Croatian | 0,017 | | 0,018 | | 0,014 | | | 0,020 | | 0,009 | -0,017 | |
| Czech | 0,066 | | 0,073 | | 0,016 | | | -0,006 | | 0,029 | -0,020 | |
| Danish | 0,058 | | 0,050 | | 0,050 | | | 0,042 | | 0,031 | 0,036 | |
| Dutch | 0,072 | | 0,033 | | 0,050 | | | 0,039 | | 0,019 | 0,023 | |
| Egyptian Arabic | 0,033 | | 0,030 | | 0,003 | | | -0,011 | | 0,033 | 0,030 | |
| English | 0,063 | | 0,042 | | 0,033 | | | 0,047 | | 0,021 | 0,057 | |
| Estonian | 0,035 | | 0,027 | | 0,017 | | | 0,024 | | 0,009 | 0,036 | |
| Farsi | 0,024 | | -0,002 | | 0,011 | | | 0,012 | | 0,008 | 0,010 | |
| Filipino | -0,016 | | 0,001 | | -0,007 | | | 0,005 | | 0,009 | 0,010 | |
| Finnish | 0,036 | | 0,029 | | 0,043 | | | 0,016 | | 0,029 | 0,013 | |
| French | 0,051 | | 0,038 | | 0,035 | | | 0,069 | | 0,024 | -0,002 | |
| Frisian | 0,065 | | 0,054 | | 0,023 | | | 0,003 | | 0,015 | 0,012 | |
| Georgian | 0,006 | | 0,001 | | 0,019 | | | 0,014 | | 0,017 | 0,015 | |
| German | -0,010 | | 0,062 | | -0,030 | | | 0,008 | | -0,011 | 0,006 | |
| Greek | 0,008 | | -0,007 | | 0,021 | | | 0,054 | | 0,001 | -0,010 | |
| Guarani | -0,017 | |  | | 0,008 | | |  | | 0,006 |  | |
| Haitian | -0,004 | | -0,082 | | 0,004 | | | -0,036 | | -0,002 | -0,033 | |
| Hebrew | 0,054 | | 0,087 | | 0,011 | | | 0,010 | | 0,019 | 0,016 | |
| Hindi | 0,026 | | 0,029 | | 0,000 | | | 0,006 | | 0,000 | -0,001 | |
| Hungarian | 0,011 | | -0,012 | | 0,001 | | | -0,008 | | -0,003 | -0,009 | |
| Icelandic | 0,024 | | 0,025 | | 0,031 | | | 0,059 | | 0,012 | -0,003 | |
| Igbo | 0,001 | |  | | 0,009 | | |  | | 0,012 |  | |
| Indonesian | 0,026 | | 0,030 | | 0,029 | | | 0,008 | | 0,019 | 0,013 | |
| Irish | 0,072 | | 0,071 | | 0,026 | | | 0,023 | | 0,030 | 0,008 | |
| Italian | 0,040 | | 0,063 | | 0,041 | | | 0,056 | | 0,029 | 0,016 | |
| Japanese | 0,000 | | 0,011 | | 0,000 | | | 0,047 | | 0,000 | 0,037 | |
| Javanese | 0,000 | | -0,008 | | 0,009 | | | 0,007 | | 0,000 | -0,001 | |
| Kazakh | 0,036 | | 0,003 | | 0,005 | | | -0,029 | | 0,002 | -0,024 | |
| Khmer | 0,075 | | 0,029 | | 0,068 | | | 0,028 | | 0,103 | 0,065 | |
| Kinyarwanda | 0,014 | |  | | 0,010 | | |  | | 0,030 |  | |
| Korean | 0,043 | | -0,021 | | 0,055 | | | 0,027 | | 0,029 | 0,010 | |
| Kyrghyz | -0,030 | | -0,010 | | 0,048 | | | 0,019 | | 0,044 | -0,002 | |
| Lao | 0,048 | |  | | 0,044 | | |  | | 0,031 |  | |
| Latvian | 0,052 | | 0,084 | | 0,070 | | | 0,069 | | 0,041 | 0,018 | |
| Lithuanian | -0,010 | | 0,055 | | -0,014 | | | 0,013 | | -0,014 | 0,022 | |
| Luxembourgish | 0,015 | | 0,020 | | -0,002 | | | 0,002 | | 0,006 | -0,004 | |
| Macedonian | 0,005 | | -0,019 | | 0,042 | | | 0,022 | | 0,018 | -0,001 | |
| Malagasy | -0,021 | | -0,013 | | -0,006 | | | 0,014 | | -0,002 | 0,021 | |
| Malay | 0,041 | | 0,030 | | 0,009 | | | 0,000 | | -0,003 | -0,001 | |
| Mongolian | -0,008 | | -0,003 | | 0,014 | | | 0,035 | | 0,006 | -0,005 | |
| Nepali | 0,009 | | 0,000 | | 0,040 | | | 0,008 | | 0,021 | -0,003 | |
| Norwegian | 0,070 | | 0,042 | | 0,035 | | | 0,035 | | 0,026 | 0,036 | |
| Nynorsk | 0,069 | | 0,035 | | 0,044 | | | 0,028 | | 0,045 | 0,024 | |
| Oromo | 0,045 | |  | | 0,007 | | |  | | -0,019 |  | |
| Pashto | 0,057 | | 0,016 | | 0,080 | | | 0,025 | | 0,050 | -0,001 | |
| Polish | 0,005 | | -0,011 | | 0,027 | | | 0,039 | | 0,033 | 0,023 | |
| Portuguese | 0,060 | | 0,049 | | 0,055 | | | 0,045 | | 0,021 | -0,014 | |
| Regional India | 0,050 | | 0,050 | | 0,032 | | | 0,039 | | 0,017 | 0,028 | |
| Romanian | 0,014 | | 0,018 | | 0,014 | | | -0,007 | | 0,023 | 0,012 | |
| Russian | -0,004 | | -0,001 | | 0,022 | | | 0,032 | | 0,017 | -0,021 | |
| Serbian | -0,007 | | -0,014 | | 0,011 | | | 0,024 | | -0,004 | -0,016 | |
| Serbo-Croatian | 0,010 | | -0,011 | | 0,002 | | | 0,012 | | 0,010 | -0,011 | |
| Shona | 0,023 | |  | | 0,002 | | |  | | 0,013 |  | |
| Sinhala | 0,046 | | 0,030 | | 0,044 | | | 0,047 | | 0,030 | 0,044 | |
| Slovak | 0,038 | | 0,024 | | 0,017 | | | 0,026 | | 0,011 | -0,009 | |
| Slovene | -0,023 | | -0,024 | | 0,029 | | | 0,014 | | 0,023 | -0,014 | |
| Somali | 0,007 | | 0,047 | | 0,006 | | | -0,033 | | -0,020 | -0,018 | |
| Spanish | 0,056 | | 0,046 | | 0,034 | | | 0,054 | | 0,023 | 0,019 | |
| Sundanese | -0,047 | | -0,021 | | 0,007 | | | 0,011 | | 0,007 | 0,014 | |
| Swahili | 0,019 | | 0,000 | | 0,007 | | | 0,029 | | 0,031 | 0,037 | |
| Swedish | 0,084 | | 0,047 | | 0,086 | | | 0,054 | | 0,066 | 0,057 | |
| Tajik | 0,020 | | 0,020 | | 0,013 | | | -0,018 | | -0,001 | -0,006 | |
| Thai | -0,020 | | 0,030 | | -0,010 | | | 0,005 | | -0,033 | 0,018 | |
| Turkish | 0,004 | | 0,009 | | 0,005 | | | 0,032 | | 0,006 | 0,026 | |
| Turkmen | 0,098 | | 0,071 | | 0,058 | | | 0,018 | | 0,081 | 0,031 | |
| Ukrainian | -0,013 | | -0,048 | | 0,051 | | | 0,056 | | 0,030 | -0,003 | |
| Urdu | 0,030 | | 0,031 | | 0,006 | | | 0,033 | | -0,001 | 0,021 | |
| Uzbek | 0,032 | | -0,039 | | 0,039 | | | -0,013 | | 0,037 | -0,024 | |
| Vietnamese | 0,048 | | 0,045 | | 0,013 | | | 0,006 | | 0,007 | -0,018 | |

Notes. The bottom table presents by language corpus the measure of linguistic gender stereotypes embedded in the Wikipedia and Common Crawl corpora. This measure captures the difference in the association of male and female words with career and family words (col.1-2), math and liberal arts words (col. 3-4) and science and arts words (col.5-6) as in the Word Embedding Association Test. Detailed descriptions of these measures are provided in Appendix A. Higher values correspond to higher association of male vs. female names with career vs. family words, math vs. liberal arts words, science vs. arts words, hence to higher gender stereotypes. The top table also provides the mean level of gender stereotypes across all language corpora in the sample, their standard deviation, the number of observations, as well as the minimum and maximum values.

**Table S2: Effect sizes. Linguistic gender stereotypes about career-family, math-liberal arts, science-arts by language corpora. Wikipedia corpora.**

|  | **Career-family ES** | | **Math-Liberal arts ES** | **Science-Arts ES** |
| --- | --- | --- | --- | --- |
| All Language Corpora | | |  |  |
| Mean | | 0,3498  (0.0477) | 0,5699  (0.0586) | 0,4508  (0.0597) |
| Std Dev. | | 0.4323 | 0.5309 | 0.5404 |
|  | |  |  |  |
| OBS. | | 82 | 82 | 82 |
| Min. | | -0.6109 | -0.6332 | -0.6335 |
| Max. | | 1.3636 | 1.6988 | 1.7514 |
|  | |  |  |  |
| By Language corpus | | |  |  |
| Afrikaans | | 0,633 | 0,614 | 0,680 |
| Albanian | | 0,308 | 0,341 | -0,052 |
| Amharic | | -0,432 | 0,286 | 0,313 |
| Arabic | | 0,673 | 0,311 | 0,459 |
| Armenian | | -0,048 | 0,089 | -0,333 |
| Azerbaijani | | -0,045 | 0,192 | 0,163 |
| Belarusian | | -0,406 | 0,538 | 0,124 |
| Bengali | | 0,187 | 0,914 | 0,660 |
| Bosnian | | 0,102 | 0,736 | -0,188 |
| Bulgarian | | 0,747 | -0,633 | -0,383 |
| Burmese | | 0,600 | 0,682 | 0,621 |
| Cantonese | | 0,319 | 0,387 | -0,633 |
| Chinese | | 0,342 | 1,105 | 0,868 |
| Croatian | | 0,281 | 0,404 | 0,423 |
| Czech | | 0,898 | 0,356 | 0,768 |
| Danish | | 0,837 | 1,205 | 1,334 |
| Dutch | | 0,791 | 1,355 | 0,703 |
| Egyptian Arabic | | 0,707 | 0,072 | 0,707 |
| English | | 0,653 | 1,156 | 0,796 |
| Estonian | | 0,669 | 0,504 | 0,388 |
| Farsi | | 0,309 | 0,519 | 0,741 |
| Filipino | | -0,192 | -0,588 | 0,595 |
| Finnish | | 0,360 | 0,887 | 0,938 |
| French | | 0,377 | 0,888 | 0,849 |
| Frisian | | 1,162 | 0,752 | 0,844 |
| Georgian | | 0,066 | 0,554 | 0,405 |
| German | | -0,269 | -0,558 | -0,337 |
| Greek | | 0,105 | 0,387 | 0,035 |
| Guarani | | -0,194 | 0,183 | 0,108 |
| Haitian | | -0,102 | 0,079 | -0,042 |
| Hebrew | | 0,628 | 0,312 | 0,682 |
| Hindi | | 0,312 | 0,014 | -0,009 |
| Hungarian | | 0,141 | 0,021 | -0,102 |
| Icelandic | | 0,486 | 1,015 | 0,457 |
| Igbo | | 0,196 | 0,845 | 0,835 |
| Indonesian | | 0,280 | 0,908 | 0,584 |
| Irish | | 0,924 | 0,535 | 0,532 |
| Italian | | 0,476 | 1,247 | 1,147 |
| Japanese | | -0,011 | 0,399 | -0,442 |
| Javanese | | -0,003 | 0,717 | 0,023 |
| Kazakh | | 0,515 | 0,145 | 0,060 |
| Khmer | | 1,364 | 1,578 | 1,628 |
| Kinyarwanda | | 0,630 | 0,162 | 0,344 |
| Korean | | 0,382 | 1,238 | 0,901 |
| Kyrghyz | | -0,381 | 0,944 | 0,921 |
| Lao | | 1,064 | 1,686 | 1,099 |
| Latvian | | 0,831 | 1,699 | 1,524 |
| Lithuanian | | -0,131 | -0,567 | -0,527 |
| Luxembourgish | | 0,390 | -0,077 | 0,189 |
| Macedonian | | 0,066 | 0,969 | 0,572 |
| Malagasy | | -0,263 | -0,119 | -0,064 |
| Malay | | 0,472 | 0,621 | -0,330 |
| Mongolian | | -0,111 | 0,320 | 0,209 |
| Nepali | | 0,124 | 1,043 | 0,632 |
| Norwegian | | 0,858 | 1,545 | 1,328 |
| Nynorsk | | 1,193 | 1,515 | 1,423 |
| Oromo | | 0,824 | 0,127 | -0,378 |
| Pashto | | 0,571 | 1,081 | 0,846 |
| Polish | | 0,061 | 0,410 | 0,703 |
| Portuguese | | 0,669 | 1,072 | 0,654 |
| Regional India | | 0,745 | 0,890 | 0,324 |
| Romanian | | 0,202 | 0,391 | 0,858 |
| Russian | | -0,044 | 0,515 | 0,522 |
| Serbian | | -0,114 | 0,346 | -0,177 |
| Serbo-Croatian | | 0,143 | 0,065 | 0,143 |
| Shona | | 0,573 | 0,117 | 0,605 |
| Sinhala | | 0,638 | 1,106 | 0,677 |
| Slovak | | 0,590 | 0,414 | 0,372 |
| Slovene | | -0,449 | 0,927 | 0,659 |
| Somali | | 0,085 | 0,226 | -0,513 |
| Spanish | | 0,543 | 0,937 | 0,945 |
| Sundanese | | -0,611 | 0,170 | 0,188 |
| Swahili | | 0,303 | 0,213 | 0,748 |
| Swedish | | 1,035 | 1,442 | 1,751 |
| Tajik | | 0,343 | 0,443 | -0,055 |
| Thai | | -0,220 | -0,293 | -0,597 |
| Turkish | | 0,042 | 0,121 | 0,166 |
| Turkmen | | 0,931 | 1,164 | 1,333 |
| Ukrainian | | -0,140 | 0,995 | 0,856 |
| Urdu | | 0,457 | 0,219 | -0,027 |
| Uzbek | | 0,534 | 0,821 | 0,883 |
| Vietnamese | | 1,107 | 0,386 | 0,315 |

Notes. The table is the analog of Table S1 for *normalized* values of linguistic gender stereotypes (effect sizes, ES), relying on corpora from Wikipedia only. More precisely, as in the Word Embedding Association Test, we consider the difference between the association of male words and female words with career-family words (resp. math-liberal arts, science-arts words) by unit of standard deviation of all male and female associations with career-family words (resp. math-liberal arts words, science-arts words). See detailed description in Appendix A.

**Table S3. Relationship between linguistic gender stereotypes and country levels of economic development and individualism.**

**Aggregate gender stereotypes among OECD vs. non-OECD countries**

|  | **Both sources** | | | **Common Crawl** | | | **Wikipedia** | | |  |
| --- | --- | --- | --- | --- | --- | --- | --- | --- | --- | --- |
|  | OECD | | non-OECD | | OECD | non-OECD | | OECD | non-OECD | |
|  | | |  | |  |  | |  |  | |
| Mean | | 0,0294  (0.0035) | 0,0126  (0.0026) | | 0,0286  (0.0035) | 0,0084  (0.0027) | | 0,0303  (0.0044) | 0,0164  (0.0032) | |
| Std Dev. | | 0.0184 | 0.0178 | | 0.0187 | 0.0189 | | 0.0232 | 0.0209 | |
|  | |  |  | |  |  | |  |  | |
| OBS. | | 28 | 47 | | 28 | 47 | | 28 | 54 | |

**Summary statistics for gender stereotypes and country levels of individualism and GNI for Panels A-D**

|  | **Obs.** | **Mean** | **Std Dev** | **Min.** | **Max.** |
| --- | --- | --- | --- | --- | --- |
| Indiv. | 67 | 40.67 | 21.13 | 12 | 91 |
| GNI | 67 | 22.6 | 24.82 | 1 | 88 |
| GS Career Wiki | 67 | 0.024 | 0.03 | -0,047 | 0,084 |
| GS Career CC | 63 | 0.02 | 0.03 | -0,048 | 0,087 |
| GS math Wiki | 67 | 0.02 | 0.021 | -0,03 | 0,096 |
| GS math CC | 63 | 0.022 | 0.021 | -0,029 | 0,069 |
| GS Science Wiki | 67 | 0.013 | 0.017 | -0,037 | 0,066 |
| GS Scince CC | 63 | 0.009 | 0.019 | -0,024 | 0,057 |
| All Wiki | 67 | 0.02 | 0.021 | -0,021 | 0,079 |
| All CC | 63 | 0.018 | 0.019 | -0,023 | 0,064 |

**PANEL A: Wikipedia Corpus. Gender stereotypes about career-family, math-liberal arts, science-arts (separately)**

|  | *Dependent Variable is linguistic gender stereotype about* | | | | | |
| --- | --- | --- | --- | --- | --- | --- |
|  | **Career-family** | **Career-family** | **Math-**  **Liberal Arts** | **Math-**  **Liberal Arts** | **Science-Arts** | **Science-Arts** |
|  |  |  |  |  |  |  |
| **Individualism** | 0.558*** |  | 0.318*** |  | 0.408*** |  |
|  | (0.103) |  | (0.118) |  | (0.113) |  |
| **GNI** |  | 0.471*** |  | 0.283** |  | 0.273** |
|  |  | (0.109) |  | (0.119) |  | (0.119) |
| Constant | -5.27e-09 | -4.44e-09 | -1.04e-09 | -5.41e-10 | -1.31e-09 | -8.14e-10 |
|  | (0.102) | (0.109) | (0.117) | (0.118) | (0.112) | (0.118) |
|  |  |  |  |  |  |  |
| Observations | 67 | 67 | 67 | 67 | 67 | 67 |
| R-squared | 0.312 | 0.222 | 0.101 | 0.080 | 0.167 | 0.074 |
|  | | |  |  |  |  |
|  | | |  |  |  |  |

**PANEL B: Common Crawl Corpus. Gender stereotypes about career-family, math-liberal arts, science-arts (separately)**

|  | *Dependent Variable is linguistic gender stereotype about* | | | | | |
| --- | --- | --- | --- | --- | --- | --- |
|  | **Career-family** | **Career-family** | **Math-**  **Liberal Arts** | **Math-**  **Liberal Arts** | **Science-Arts** | **Science-Arts** |
|  |  |  |  |  |  |  |
| **Individualism** | 0.598*** |  | 0.365*** |  | 0.386*** |  |
|  | (0.103) |  | (0.119) |  | (0.118) |  |
| **GNI** |  | 0.444*** |  | 0.260** |  | 0.311** |
|  |  | (0.115) |  | (0.124) |  | (0.122) |
| Constant | -3.93e-09 | -4.28e-09 | -3.73e-09 | -3.95e-09 | -2.72e-09 | -2.95e-09 |
|  | (0.102) | (0.114) | (0.118) | (0.123) | (0.117) | (0.121) |
|  |  |  |  |  |  |  |
| Observations | 63 | 63 | 63 | 63 | 63 | 63 |
| R-squared | 0.358 | 0.197 | 0.133 | 0.068 | 0.149 | 0.097 |
|  | | |  |  |  |  |

| **PANEL C: Aggregate gender stereotypes. Wikipedia and Common Crawl Corpora.**   \|  \| *Dependent Variable is linguistic gender stereotypes embedded in* \| \| \| \| \| \| \| --- \| --- \| --- \| --- \| --- \| --- \| --- \| \|  \| **Wikipedia** \| **Wikipedia** \| **CC** \| **CC** \| **Wikipedia and CC** \| **Wikipedia and CC** \| \|  \|  \|  \|  \|  \|  \|  \| \| **Individualism** \| 0.565*** \|  \| 0.660*** \|  \| 0.665*** \|  \| \|  \| (0.102) \|  \| (0.0962) \|  \| (0.0956) \|  \| \| **GNI** \|  \| 0.465*** \|  \| 0.493*** \|  \| 0.538*** \| \|  \|  \| (0.110) \|  \| (0.111) \|  \| (0.108) \| \| Constant \| -2.91e-09 \| -2.08e-09 \| 4.73e-09 \| 4.33e-09 \| 1.17e-10 \| -2.75e-10 \| \|  \| (0.102) \| (0.109) \| (0.0955) \| (0.111) \| (0.0948) \| (0.107) \| \|  \|  \|  \|  \|  \|  \|  \| \| Observations \| 67 \| 67 \| 63 \| 63 \| 63 \| 63 \| \| R-squared \| 0.319 \| 0.216 \| 0.435 \| 0.243 \| 0.443 \| 0.290 \| |
| --- | --- | --- | --- | --- | --- | --- | --- | --- | --- | --- | --- | --- | --- | --- | --- | --- | --- | --- | --- | --- | --- | --- | --- | --- | --- | --- | --- | --- | --- | --- | --- | --- | --- | --- | --- | --- | --- | --- | --- | --- | --- | --- | --- | --- | --- | --- | --- | --- | --- | --- | --- | --- | --- | --- | --- | --- | --- | --- | --- | --- | --- | --- | --- | --- | --- | --- | --- | --- | --- | --- | --- | --- | --- | --- | --- | --- | --- | --- | --- | --- | --- | --- | --- | --- |
|  |

**PANEL D: Regression of aggregate gender stereotype on individualism and economic development simultaneously. Wikipedia and Common Crawl Corpora.**

|  | *Dependent Variable is linguistic gender stereotypes embedded in* | | |
| --- | --- | --- | --- |
|  | **Wikipedia** | **CC** | **Wikipedia and CC** |
|  |  |  |  |
| **Individualism** | 0.486*** | 0.673*** | 0.606*** |
|  | (0.152) | (0.149) | (0.148) |
| **GNI** | 0.107 | -0.0182 | 0.0780 |
|  | (0.152) | (0.149) | (0.148) |
| Constant | -2.73e-09 | 4.73e-09 | 8.69e-11 |
|  | (0.102) | (0.0962) | (0.0954) |
|  |  |  |  |
| Observations | 67 | 63 | 63 |
| R-squared | 0.324 | 0.435 | 0.445 |

Notes. The top table presents measures of aggregate gender stereotypes for OECD and non-OECD countries separately, the next table presents summary statistics for the variables in Panels A-D, while Panels A-D present estimates of linear regressions of country-level gender stereotypes, based on semantic similarity, on country levels of individualism (Hofstede) and economic development (Gross National Income, GNI). Panel A (resp. Panel B) considers separately gender stereotypes about career-family, math-liberal arts and science-arts embedded in the Wikipedia (resp. Common Crawl) corpora. Panels C and D consider aggregate gender stereotypes, relying on a weighted average of the three gender stereotypes (see Appendix A). Panel C considers simple linear regressions like Panels A and B, while Panel D considers linear regressions that include individualism and GNI simultaneously as explanatory variables. Our measures of linguistic gender stereotypes rely on the Word Embedding Association Test (see Appendix A). The sample is restricted to countries with available data for Hofstede's measure of individualism and GNI. All variables are standardized on the regression sample. Standard errors in parentheses ^***^ *p<0.01,* ^**^ *p<0.05,* ^*^ *p<0.1*

**Table S4. Single Category linguistic stereotypical associations: Relationship with country levels of economic development and individualism**

**Panel A: Single Category gender stereotypical associations related to career and family (Wikipedia source)**

|  | |  |  |  |  |  |  |  |  |
| --- | --- | --- | --- | --- | --- | --- | --- | --- | --- |
|  | CAREER | | FAMILY | MALE | FEMALE | CAREER | FAMILY | MALE | FEMALE |
|  |  | |  |  |  |  |  |  |  |
| **Indiv.** | 0.492*** | | -0.280** | -0.120 | -0.425*** |  |  |  |  |
|  | (0.108) | | (0.119) | (0.123) | (0.112) |  |  |  |  |
| **GNI** |  | |  |  |  | 0.384*** | -0.261** | 0.0249 | -0.245** |
|  |  | |  |  |  | (0.115) | (0.120) | (0.124) | (0.120) |
| Constant | 3.75e-09 | | 4.76e-09 | 4.79e-09 | -1.60e-09 | 4.44e-09 | 4.31e-09 | 4.81e-09 | -2.06e-09 |
|  | (0.107) | | (0.118) | (0.122) | (0.111) | (0.114) | (0.119) | (0.123) | (0.119) |
|  |  | |  |  |  |  |  |  |  |
| Observations | 67 | | 67 | 67 | 67 | 67 | 67 | 67 | 67 |
| R-squared | 0.242 | | 0.078 | 0.014 | 0.180 | 0.147 | 0.068 | 0.001 | 0.060 |

**Panel B: Single Category gender stereotypical associations related to math or science and liberal arts or arts (Wikipedia source)**

|  | |  | |  |  | | |  | |  |  |  | |  |
| --- | --- | --- | --- | --- | --- | --- | --- | --- | --- | --- | --- | --- | --- | --- |
|  | MATH  &SCIENCE | | LIB. ARTS  &ARTS | | | MALE | FEMALE | | MATH  &SCIENCE | | LIB. ARTS  &ARTS | | MALE | FEMALE |
|  |  | |  | | |  |  | |  | |  | |  |  |
| **Indiv.** | 0.256** | | -0.180 | | | 0.0421 | -0.153 | |  | |  | |  |  |
|  | (0.120) | | (0.122) | | | (0.124) | (0.123) | |  | |  | |  |  |
| **GNI** |  | |  | | |  |  | | 0.304** | | -0.0428 | | 0.126 | -0.0277 |
|  |  | |  | | |  |  | | (0.118) | | (0.124) | | (0.123) | (0.124) |
| Constant | 2.18e-09 | | -4.95e-09 | | | 6.34e-10 | -5.08e-10 | | 2.70e-09 | | -5.05e-09 | | 8.31e-10 | -5.84e-10 |
|  | (0.119) | | (0.121) | | | (0.123) | (0.122) | | (0.117) | | (0.123) | | (0.122) | (0.123) |
|  |  | |  | | |  |  | |  | |  | |  |  |
| Observations | 67 | | 67 | | | 67 | 67 | | 67 | | 67 | | 67 | 67 |
| R-squared | 0.066 | | 0.032 | | | 0.002 | 0.024 | | 0.092 | | 0.002 | | 0.016 | 0.001 |

**Panel C: Single Category gender stereotypical associations related to career/math/science and family/liberal arts/arts (Wikipedia source).**

|  | |  | |  |  |  |  |  |  |  |
| --- | --- | --- | --- | --- | --- | --- | --- | --- | --- | --- |
|  | CAREER, MATH and SCIENCE | | FAMILY, LIBERAL ARTS and ARTS | | MALE | FEMALE | CAREER, MATH and SCIENCE | FAMILY, LIBERAL ARTS and ARTS | MALE | FEMALE |
|  |  | |  | |  |  |  |  |  |  |
| **Indiv.** | 0.437*** | | -0.287** | | -0.0575 | -0.357*** |  |  |  |  |
|  | (0.112) | | (0.119) | | (0.124) | (0.116) |  |  |  |  |
| **GNI** |  | |  | |  |  | 0.392*** | -0.207* | 0.0763 | -0.178 |
|  |  | |  | |  |  | (0.114) | (0.121) | (0.124) | (0.122) |
| Constant | 1.40e-09 | | -1.83e-09 | | 8.36e-10 | 4.69e-10 | 2.09e-09 | -2.20e-09 | 9.38e-10 | 1.22e-10 |
|  | (0.111) | | (0.118) | | (0.123) | (0.115) | (0.113) | (0.120) | (0.123) | (0.121) |
|  |  | |  | |  |  |  |  |  |  |
| Obs. | 67 | | 67 | | 67 | 67 | 67 | 67 | 67 | 67 |
| R-squared | 0.191 | | 0.082 | | 0.003 | 0.127 | 0.154 | 0.043 | 0.006 | 0.032 |

**Panel D: Single Category gender stereotypical associations related to career/math/science and family/liberal arts/arts. Common Crawl**

|  | |  | |  |  |  |  |  |  |  |
| --- | --- | --- | --- | --- | --- | --- | --- | --- | --- | --- |
|  | CAREER, MATH and SCIENCE | | FAMILY, LIBERAL ARTS and ARTS | | MALE | FEMALE | CAREER, MATH and SCIENCE | FAMILY, LIBERAL ARTS and ARTS | MALE | FEMALE |
|  |  | |  | |  |  |  |  |  |  |
| **Indiv.** | 0.365*** | | 0.0161 | | -0.0730 | -0.454*** |  |  |  |  |
|  | (0.119) | | (0.128) | | (0.128) | (0.114) |  |  |  |  |
| **GNI** |  | |  | |  |  | 0.303** | 0.0480 | 0.0806 | -0.231* |
|  |  | |  | |  |  | (0.122) | (0.128) | (0.128) | (0.125) |
| Constant | 1.76e-09 | | 9.87e-10 | | 8.35e-10 | -5.05e-09 | 1.55e-09 | 9.83e-10 | 8.99e-10 | -4.76e-09 |
|  | (0.118) | | (0.127) | | (0.127) | (0.113) | (0.121) | (0.127) | (0.127) | (0.124) |
|  |  | |  | |  |  |  |  |  |  |
| Obs. | 63 | | 63 | | 63 | 63 | 63 | 63 | 63 | 63 |
| R-squared | 0.133 | | 0.000 | | 0.005 | 0.206 | 0.092 | 0.002 | 0.007 | 0.053 |

Notes. The table presents estimates of linear regressions of country-level single category gender stereotypical linguistic associations on country levels of individualism (Hofstede individualism) and economic development (Gross National Income, GNI). Measures of single-category associations rely on the single-category Word Embedding Association Test and are detailed in Appendix A. Panel A considers separately the association of (i) career words with male versus female words, (ii) family words with male versus female words, (iii) male words with career versus family words and (iv) female words with career versus family words. Panel B considers separately associations of (i) math or science and (ii) liberal arts or arts words with male versus female words as well as associations of (iii) male and (iv) female words with math or science versus liberal arts or arts words. Panel C considers career, math and science words together as well as family, liberal arts and arts words by relying on a weighted average of the three gender stereotypes (see Appendix A). It considers associations of (i) career, math and science words and (ii) family, liberal arts and arts words with male versus female words as well as associations of (iii) male and (iv) female words with career, math and science words versus family, liberal arts and arts words (See Appendix A for the measure of aggregate stereotype). The sample is restricted to countries with available date for Hofstede's measure of individualism and GNI and all variables are standardized on the regression sample. Standard errors in parentheses ^***^ *p<0.01,* ^**^ *p<0.05,* ^*^ *p<0.1*

**Table S5. Robustness to other stimuli: stimuli adopted in (12) about work versus home and about science versus arts**

**PANEL A: Descriptive statistics (Wikipedia source)**

|  | **Career-Family** | | **Science-Arts** | |
| --- | --- | --- | --- | --- |
|  | Diff. | Effect size | Diff. | Effect size |
|  |  |  |  |  |
| All Corpora | 0,0260  (0.0036) | 0,3823  (0.0485) | 0,0159  (0.0029) | 0,3586  (0.0586) |
| Std Dev. | 0.0323 | 0.4394 | 0.0266 | 0.5306 |
|  |  |  |  |  |
| OBS. | 82 | 82 | 82 | 82 |
| Min. | -0.0428 | -0.5384 | -0.0275 | -0.6547 |
| Max. | 0.1288 | 1.3974 | 0.1184 | 1.6341 |

**PANEL B: Relations of linguistic gender stereotypes GS across stimuli (Wikipedia source)**

|  | GS  **Career-Family**  **new stimuli**  Wikipedia | GS  **Career-Family**  **main stimuli**  Wikipedia | GS  **Science-arts new stimuli**  Wikipedia | GS  **Science-arts**  **main stimuli**  Wikipedia |
| --- | --- | --- | --- | --- |
| GS Career-Family (new stimuli) | 1 |  |  |  |
| GS Career Family (main stimuli) | 0,799*** | 1 |  |  |
| GS Science-Arts (new stimuli) | 0,553*** | 0,576*** | 1 |  |
| GS Science-Arts (main stimuli) | 0,464*** | 0,510*** | 0,738*** | 1 |

**PANEL C: Relationship with country levels of economic development and individualism**

|  | **Career-**  **Family**  in WIKI | **Science-**  **Arts**  in WIKI | **Career-**  **Family**  in CC | **Science-**  **Arts**  in CC | **Career-**  **Family**  in WIKI | **Science-**  **Arts**  in WIKI | **Career-**  **Family**  in CC | **Science-**  **Arts**  in CC |
| --- | --- | --- | --- | --- | --- | --- | --- | --- |
|  |  |  |  |  |  |  |  |  |
| **Individualism** | 0.347*** | 0.379*** | 0.342*** | 0.407*** |  |  |  |  |
|  | (0.116) | (0.115) | (0.120) | (0.117) |  |  |  |  |
| **GNI** |  |  |  |  | 0.341*** | 0.346*** | 0.270** | 0.230* |
|  |  |  |  |  | (0.117) | (0.116) | (0.123) | (0.125) |
| Constant | -3.59e-09 | 8.44e-10 | -2.03e-09 | -2.22e-09 | -3.00e-09 | 1.45e-09 | -2.24e-09 | -2.48e-09 |
|  | (0.115) | (0.114) | (0.119) | (0.116) | (0.116) | (0.116) | (0.122) | (0.124) |
|  |  |  |  |  |  |  |  |  |
| Observations | 67 | 67 | 63 | 63 | 67 | 67 | 63 | 63 |
| R-squared | 0.121 | 0.144 | 0.117 | 0.165 | 0.116 | 0.120 | 0.073 | 0.053 |

Notes. The table considers gender stereotypes measured through linguistic associations embedded in Wikipedia and Common Crawl corpora, relying on alternative stimuli than in the (main) setting adopted in previous tables. These alternative stimuli are related to work and home, and to science and arts. They are the same as in (12) and described in detail in Appendix A. Panel A (resp. Panel B, C) is the analog of Table S1 (resp. Table S3, Table S4) for these new stimuli. See the Notes of these tables for more details. Panel A describes the measures of linguistic gender stereotypes embedded in Wikipedia corpora for these alternative stimuli. Panel B considers the correlation between gender stereotypes embedded in the Wikipedia corpora and in the Common Crawl corpora, and between stereotypes measured with the stimuli adopted in the main setting and in this new setting. Panel C analyzes the relation between gender stereotypes and country levels of individualism (Hofstede) and Gross National Income (GNI). Measures of linguistic associations rely on the Word Embedding Association Test. All variables are standardized on the regression sample. Standard errors in parentheses ^***^ *p<0.01,* ^**^ *p<0.05,* ^*^ *p<0.1*

**Table S6. Robustness (of the relation between gender stereotypes and country individualism and economic development) to other measures of economic development and individualism**

**PANEL A: Measure of collectivism from the Globe survey, Gross Domestic Product and Human Development Index**

|  | | Career-Family Stereotypes (WIKI) | Math-Liberal arts Stereotypes (WIKI) | Science-Arts Stereotypes (WIKI) | All three Stereotypes (WIKI) | All three Stereotypes (CC) | All three stereotypes (WIKI & CC) |
| --- | --- | --- | --- | --- | --- | --- | --- |
| Collectivism  (M, GLOBE2004,  40 obs.) | | 0,562*** | 0,394** | 0,364** | 0,556*** | 0,528*** | 0,599*** |
| GDP (2000-2020, 78 obs.) | | 0,443*** | 0,261** | 0,272** | 0,439*** | 0,441*** | 0,492*** |
| HDI (2005-2020, 80 obs.) | | 0,333*** | 0,257** | 0,202 | 0,345*** | 0,355*** | 0,399*** |
|  |  | |  |  |  |  |  |

**PANEL B: Historical measures of economic development**

|  | Career-Family Stereotypes (WIKI) | Math-Liberal arts Stereotypes (WIKI) | Science-Arts Stereotypes (WIKI) | All three Stereotypes (WIKI) | All three Stereotypes (CC) | All three stereotypes (WIKI & CC) |
| --- | --- | --- | --- | --- | --- | --- |
| GDP 1960  (36 obs.) | 0,556*** | 0,403** | 0,458*** | 0,551*** | 0,516*** | 0,571*** |
| GDP 1970  (43 obs.) | 0,482*** | 0,273* | 0,331** | 0,449*** | 0,481*** | 0,492*** |
| GDP 1980  (45 obs.) | 0,453*** | 0,295* | 0,341** | 0,445*** | 0,472*** | 0,493*** |
| GDP 1990  (65 obs.) | 0,436*** | 0,271** | 0,273** | 0,438*** | 0,503*** | 0,515*** |

Notes. The table presents estimates of correlation coefficients (or equivalently of regression coefficients with standardized variables) between gender stereotypes embedded in Wikipedia and Common Crawl corpora and measures of individualism and economic development. We consider gender stereotypes about Career-family, math-liberal arts, science-arts as well as a measure of gender stereotypes that collapses the three (see Appendix A). In Panel A, we consider as alternative measures of individualism the opposite of the measure of collectivism in practice from the Globe survey (2004), and we consider as alternative measures of economic development the Gross Domestic Product (considering averages of GDP measures between 2000 and 2020) and the Human Development Index (considering averages of HDI measures between 2005 and 2020). In Panel B, we consider historical measures, with measures of Gross Domestic Product in 1960, 1970, 1980, and 1990. The letter M denotes that the opposite of the measure is considered, so that higher values correspond to higher levels of individualism or economic development. The sample is restricted to countries with available data for Hofstede's measure of individualism and Gross National Income. Measures and data sources are described in Appendix B.^***^ *p<0.01,* ^**^ *p<0.05,* ^*^ *p<0.1*

**Table S7. Relation of language gender stereotypes with country-level measures of gender equality, and ecological stress. Simple Regressions**

**PANEL A: Gender Equality. Simple regressions**

|  | Career Stereotypes (WIKI) | Math Stereotypes (WIKI) | Science Stereotypes (WIKI) | All Stereotypes (WIKI) | All Stereotypes (WIKI & CC) |
| --- | --- | --- | --- | --- | --- |
| **GGI** | 0.241* | 0.294** | 0.299** | 0.306** | 0.303** |
|  | (0.121) | (0.119) | (0.119) | (0.119) | (0.122) |
| **Female**  **labor force** | 0.0495 | 0.153 | 0.0804 | 0.0903 | 0.0924 |
|  | (0.125) | (0.124) | (0.125) | (0.124) | (0.129) |
| **Women**  **fertility rate** | -0.0721 | -0.258** | 0.00888 | -0.114 | -0.0498 |
|  | (0.124) | (0.120) | (0.124) | (0.123) | (0.128) |
| **Value Politics**  **(Gender Equality)** | 0.517*** | 0.449*** | 0.420*** | 0.571*** | 0.622*** |
|  | (0.118) | (0.123) | (0.125) | (0.113) | (0.111) |
| **Value Education (Gender Equality)** | 0.436*** | 0.366*** | 0.377*** | 0.483*** | 0.520*** |
|  | (0.124) | (0.128) | (0.127) | (0.120) | (0.121) |
| **Value Jobs**  **(Gender Equality)** | 0.430*** | 0.450*** | 0.338** | 0.491*** | 0.544*** |
|  | (0.124) | (0.123) | (0.129) | (0.120) | (0.119) |
| **Value index**  **(Gender Equality)** | 0.451*** | 0.427*** | 0.316** | 0.496*** | 0.546*** |
|  | (0.123) | (0.124) | (0.130) | (0.119) | (0.118) |

**PANEL B: Ecological Stress. Simple regressions**

|  | Career Stereotypes (WIKI) | | Math Stereotypes (WIKI) | Science Stereotypes (WIKI) | All Stereotypes (WIKI) | All Stereotypes (WIKI & CC) |
| --- | --- | --- | --- | --- | --- | --- |
| **Calories (hist.)** | 0.315* | | 0.371** | 0.383** | 0.387** | 0.392** |
|  | (0.160) | | (0.157) | (0.156) | (0.156) | (0.158) |
| **Calories (cont.)** | 0.389** | | 0.309* | 0.384** | 0.424*** | 0.475*** |
|  | (0.156) | | (0.161) | (0.156) | (0.153) | (0.151) |
| **Disease (hist.)** | -0.292* | | -0.404** | -0.435*** | -0.390** | -0.346** |
|  | (0.162) | | (0.155) | (0.152) | (0.156) | (0.161) |
| **Disease (cont.)** | -0.284* | | -0.201 | -0.239 | -0.295* | -0.283* |
|  | (0.162) | | (0.166) | (0.164) | (0.162) | (0.165) |
|  | |  |  |  |  |  |

Notes. The table presents estimates of linear regressions of country-level gender stereotypical linguistic associations in the Wikipedia and Common Crawl corpora on various country-level measures of gender equality (Panel A) and ecological stress (Panel B). Concerning measures of gender equality, we consider the Gender Gap Index (GGI) which is an index related to the level of gender equality in practice and more specific measures such as country levels of female labor force participation and fertility rate. We also consider four measures of gender equality in values (regarding politics, education, occupations, and an index) relying on the World Value Survey (Wave 7, 2017-2020). Concerning measures of ecological stress, we consider the same measures as in (15), *i.e.*, lack of nutrition and disease, using both historical estimates (hist.) and contemporary estimates (cont.). Concerning stereotypes, we consider gender stereotypes, measured by the Word Embedding Association Test, about career-family (col. 1), math-liberal arts (col.2), science-arts (col. 3), and about the three in col. 4 (Wikipedia source only) and col. 5 (both sources). All variables are standardized on the regression sample. Standard errors in parentheses ^***^ *p<0.01,* ^**^ *p<0.05,* ^*^ *p<0.1*

**Table S8. Relation of language gender stereotypes with country-level measures of gender equality, and ecological stress. Controlling for levels of individualism or economic development**

**PANEL A: Gender Gap Index. Controlling for GNI or individualism**

|  | **Career-Family, Math-liberal arts, Science-art** | | **Career-Family** | | **Math-liberal arts** | | **Science-Art** | |
| --- | --- | --- | --- | --- | --- | --- | --- | --- |
| VARIABLES | (1) | (2) | (3) | (4) | (5) | (6) | (7) | (8) |
|  |  |  |  |  |  |  |  |  |
| **GGI** | -0.0226 | -0.0514 | -0.0790 | -0.102 | 0.166 | 0.126 | -0.0308 | -0.0368 |
|  | (0.135) | (0.112) | (0.138) | (0.116) | (0.151) | (0.139) | (0.147) | (0.133) |
| **GNI** | 0.551*** |  | 0.554*** |  | 0.199 |  | 0.407*** |  |
|  | (0.135) |  | (0.138) |  | (0.151) |  | (0.147) |  |
| **Indiv.** |  | 0.692*** |  | 0.682*** |  | 0.308** |  | 0.481*** |
|  |  | (0.112) |  | (0.116) |  | (0.139) |  | (0.133) |
|  |  |  |  |  |  |  |  |  |
| Constant | -3.40e-10 | -0 | 2.41e-09 | 2.74e-09 | -2.3e-09 | -2.26e-09 | -5.65e-09 | -5.38e-09 |
|  | (0.108) | (0.0954) | (0.110) | (0.0988) | (0.121) | (0.118) | (0.118) | (0.113) |
|  |  |  |  |  |  |  |  |  |
| Observations | 63 | 63 | 63 | 63 | 63 | 63 | 63 | 63 |
| R-squared | 0.290 | 0.445 | 0.261 | 0.405 | 0.106 | 0.150 | 0.152 | 0.215 |

**PANEL B: Gender Equality in Values. Controlling for GNI or individualism**

|  | **Career-Family, Math-liberal arts, Science-art** | | **Career-Family** | | **Math-liberal arts** | | **Science-Art** | |
| --- | --- | --- | --- | --- | --- | --- | --- | --- |
| VARIABLES | (1) | (2) | (3) | (4) | (5) | (6) | (7) | (8) |
|  |  |  |  |  |  |  |  |  |
| **Equality Values** | 0.0196 | 0.101 | 0.00614 | 0.0207 | 0.306 | 0.409** | -0.316 | -0.108 |
|  | (0.175) | (0.154) | (0.194) | (0.166) | (0.205) | (0.184) | (0.192) | (0.179) |
| **GNI** | 0.659*** |  | 0.572*** |  | 0.226 |  | 0.813*** |  |
|  | (0.175) |  | (0.194) |  | (0.205) |  | (0.192) |  |
| **Indiv.** |  | 0.606*** |  | 0.602*** |  | 0.105 |  | 0.600*** |
|  |  | (0.154) |  | (0.166) |  | (0.184) |  | (0.179) |
|  |  |  |  |  |  |  |  |  |
| Constant | -4.99e-09 | -4.73e-09 | -5.22e-09 | -5.89e-09 | 2.25e-09 | 3.54e-09 | -5.63e-09 | -3.58e-09 |
|  | (0.104) | (0.103) | (0.116) | (0.111) | (0.122) | (0.123) | (0.114) | (0.120) |
|  |  |  |  |  |  |  |  |  |
| Observations | 52 | 52 | 52 | 52 | 52 | 52 | 52 | 52 |
| R-squared | 0.455 | 0.467 | 0.333 | 0.381 | 0.255 | 0.241 | 0.350 | 0.276 |

**PANEL C: Ecological Stress. Controlling for GNI or individualism**

|  |  | | |  | |  | |  |  |  |  |
| --- | --- | --- | --- | --- | --- | --- | --- | --- | --- | --- | --- |
|  | *Dependent Variable is linguistic aggregate gender stereotypes*  *(*Career-Family, Math-liberal arts, Science-art) | | | | | | | | | | |
|  | (1) | | (2) | | (3) | | (4) | | (5) | (6) |  |
|  | |  |  | |  | |  | |  |  |  |
| **Individualism** | | 0.777*** |  | | 0.638*** | |  | | 0.756*** |  |  |
|  | | (0.169) |  | | (0.159) | |  | | (0.157) |  |  |
| **GNI** | |  | 0.664*** | |  | | 0.595*** | |  | 0.821*** |  |
|  | |  | (0.160) | |  | | (0.162) | |  | (0.176) |  |
| **Calories (hist)** | | -0.130 | 0.00247 | |  | |  | |  |  |  |
|  | | (0.169) | (0.160) | |  | |  | |  |  |  |
| **Calories (cont)** | |  |  | | 0.0849 | | 0.116 | |  |  |  |
|  | |  |  | | (0.159) | | (0.162) | |  |  |  |
| **Disease (hist)** | |  |  | |  | |  | | 0.109 | 0.224 |  |
|  | |  |  | |  | |  | | (0.157) | (0.176) |  |
| Constant | | 6.74e-10 | 4.31e-09 | | 1.74e-09 | | 4.86e-09 | | 6.78e-10 | 4.23e-09 |  |
|  | | (0.123) | (0.128) | | (0.124) | | (0.127) | | (0.123) | (0.125) |  |
|  | |  |  | |  | |  | |  |  |  |
| Observations | | 36 | 36 | | 36 | | 36 | | 36 | 36 |  |
| R-squared | | 0.485 | 0.443 | | 0.480 | | 0.451 | | 0.483 | 0.469 |  |

Notes. The table presents estimates of linear regressions of country-level gender stereotypes (based on semantic similarity in both Wikipedia and Common Crawl corpora) on country level measures of gender equality (Gender Gap Index in Panel A and Gender equality in Values, from the World Value Survey, in Panel B) and measures of ecological stress (lack of nutrition and disease, using both historical estimates (hist.) and contemporary estimates (cont.) in Panel C), controlling for country levels of individualism (Hofstede's measure of individualism) and economic development (Gross National Income, GNI). Language gender stereotypes are about career, math and science and measured as in (5) by the Word Embedding Association Test. In Panels A and B, Columns 3-8 consider each stereotype separately: male-career/female-family stereotypes in columns 3-4, male-math/female-liberal arts stereotypes in columns 5-6 and male-science/female-arts stereotypes in columns 7-8. Col. 1 -2 consider the three stereotypes together as does Panel C. The sample is restricted to countries with available data for Hofstede's measure of individualism and GNI and all variables are standardized on the regression sample. Standard errors in parentheses ^***^ *p<0.01,* ^**^ *p<0.05,* ^*^ *p<0.1*

**Table S9. Robustness (of the relation between gender stereotypes and country individualism and economic development) to normalized measures of stereotypes (Effect sizes)**

**PANEL A: Wikipedia**

|  | **ES**  **Career-Family** | **ES**  **Career-Family** | **ES**  **Math-Lib. Arts** | **ES**  **Math-Lib. Arts** | **ES**  **Science-Arts** | **ES**  **Science-Arts** |
| --- | --- | --- | --- | --- | --- | --- |
|  |  |  |  |  |  |  |
| **Individualism** | 0.491*** |  | 0.339*** |  | 0.461*** |  |
|  | (0.108) |  | (0.117) |  | (0.110) |  |
| **GNI** |  | 0.430*** |  | 0.336*** |  | 0.341*** |
|  |  | (0.112) |  | (0.117) |  | (0.117) |
| Cstt | 1.78e-09 | 2.54e-09 | -2.04e-09 | -1.45e-09 | -3.02e-10 | 3.12e-10 |
|  | (0.107) | (0.111) | (0.116) | (0.116) | (0.109) | (0.116) |
|  |  |  |  |  |  |  |
| Obs | 67 | 67 | 67 | 67 | 67 | 67 |
| R-sq | 0.241 | 0.185 | 0.115 | 0.113 | 0.213 | 0.116 |

**PANEL B: Common Crawl**

|  | **ES**  **Career-Family** | **ES**  **Career-Family** | **ES**  **Math-Lib. Arts** | **ES**  **Math-Lib. Arts** | **ES**  **Science-Arts** | **ES**  **Science-Arts** |
| --- | --- | --- | --- | --- | --- | --- |
|  |  |  |  |  |  |  |
| **Individualism** | 0.574*** |  | 0.346*** |  | 0.395*** |  |
|  | (0.105) |  | (0.120) |  | (0.118) |  |
| **GNI** |  | 0.435*** |  | 0.301** |  | 0.322** |
|  |  | (0.115) |  | (0.122) |  | (0.121) |
| Cstt | 4.05e-09 | 3.71e-09 | 3.78e-10 | 1.78e-10 | 4.40e-09 | 4.17e-09 |
|  | (0.104) | (0.114) | (0.119) | (0.121) | (0.117) | (0.120) |
|  |  |  |  |  |  |  |
| Obs | 63 | 63 | 63 | 63 | 63 | 63 |
| R-sq | 0.329 | 0.189 | 0.120 | 0.091 | 0.156 | 0.104 |

Notes. The table is the analog of Table S4 for normalized measures of gender stereotypes, *i.e.*, effect sizes instead of our main measure of gender stereotypes GS. See details in Appendix A about these measures of stereotypes that both rely on the Word Embedding Association Test. The table presents estimates of linear regressions of country-level gender stereotypes, based on semantic similarity, on country levels of individualism (Hofstede individualism) and economic development (Gross National Income, GNI). Both panels consider gender stereotypes about career-family, math-liberal arts and science-arts embedded in the Wikipedia (Panel A) and Common Crawl (Panel B) corpora. The sample is restricted to countries with available data for individualism and GNI. All variables are standardized on the regression sample. Standard errors in parentheses ^***^ *p<0.01,* ^**^ *p<0.05,* ^*^ *p<0.1*

**Table S10. Additional robustness checks (of the relation between gender stereotypes and country individualism and economic development)**

**PANEL A: Individualism**

|  | Proportion | | Absolute | | Most Speakers | | Major contributors | | OECD  countries | | Control for genderedness | | Control for continent and language family |  |
| --- | --- | --- | --- | --- | --- | --- | --- | --- | --- | --- | --- | --- | --- | --- |
|  | | | | |  | |  | |  | |  | |  |  |
| **Indiv.** | 0.630*** | | 0.642*** | | 0.664*** | | 0.655*** | | 0.569*** | | 0.595*** | | 0.767*** |  |
|  | (0.0994) | | (0.118) | | (0.0949) | | (0.114) | | (0.161) | | (0.131) | | (0.115) |  |
|  |  | |  | |  | |  | |  | |  | |  |  |
| Constant | -4.06e-09 | | -3.16e-10 | | -1.06e-09 | | 3.16e-09 | | -3.38e-09 | | -0.179 | | 0.128 |  |
|  | (0.0986) | | (0.117) | | (0.0942) | | (0.113) | | (0.158) | | (0.215) | | (0.450) |  |
|  |  | |  | |  | |  | |  | |  | |  |  |
| Obs. | 63 | | 44 | | 64 | | 46 | | 28 | | 39 | | 62 |  |
| R-sq. | 0.397 | | 0.413 | | 0.441 | | 0.429 | | 0.324 | | 0.401 | | 0.611 |  |
|  | |  | |  | |  | |  | |  | |  | |  |

**PANEL B: GNI**

|  | Proportion | Absolute | | Most Speakers | | Major contributors | | OECD countries | | Control for  genderedness | | | Control for continent and language family |
| --- | --- | --- | --- | --- | --- | --- | --- | --- | --- | --- | --- | --- | --- |
|  |  |  | |  | |  | |  | |  | | |  |
| **GNI** | 0.499*** | 0.527*** | | 0.539*** | | 0.616*** | | 0.380** | | 0.633*** | | | 0.605*** |
|  | (0.111) | (0.131) | | (0.107) | | (0.119) | | (0.181) | | (0.125) | | | (0.134) |
|  |  |  | |  | |  | |  | |  | | |  |
| Constant | 5.09e-10 | -5.27e-10 | | -1.99e-09 | | 1.66e-09 | | -2.55e-09 | | -0.208 | | | 0.158 |
|  | (0.110) | (0.130) | | (0.106) | | (0.117) | | (0.178) | | (0.205) | | | (0.529) |
|  |  |  | |  | |  | |  | |  | | |  |
| Obs. | 63 | 44 | | 64 | | 46 | | 28 | | 39 | | | 62 |
| R-sq. | 0.249 | 0.277 | | 0.291 | | 0.380 | | 0.145 | | 0.451 | | | 0.466 |
|  |  | |  | |  | |  | |  | |  |  | |

Notes. The table presents estimates of linear regressions of country-level gender stereotypical linguistic associations on levels of individualism (Hofstede, Panel A) and Gross National Income, (GNI, Panel B) for various specifications. We consider aggregate gender stereotypes (career-family, math-liberal arts and science-arts) captured in corpora from both Wikipedia and Common Crawl. Concerning the various specifications, col. 1-3 correspond to different matches between language corpora and levels of individualism and GNI. In col.1 (Proportion), we consider for each language corpus the weighted average of the levels of individualism and of GNI of the countries contributing to the corpus, according to their relative contribution. In col.2 (Absolute), we restrict the sample to countries whose contribution to the language corpus is greater than 50%. In col. 3, we consider for each language corpus the levels of individualism and of GNI of the country with the highest number of speakers of the given language. Col. 4-5 consider the restriction of the sample of corpora (i) to the largest corpora in the Wikipedia project (top 50, more than 0.01% of the total Wikipedia, col.4) and (ii) to corpora of OECD countries (col.5). In col. 6, we add in the regression a control for language genderedness and in col. 7 a control for continent and language family (see Appendix B). The different specifications are detailed in Appendix A. The sample is restricted to countries with available data for individualism and GNI. All variables are standardized on the regression sample. Standard errors in parentheses ^***^ *p<0.01,* ^**^ *p<0.05,* ^*^ *p<0.1*

**Table S11. Robustness (of the relation between gender stereotypes and country individualism and economic development) to other translation tool (ChatGPT). Wikipedia corpus.**

|  |  | |  | | |  | |  | |  |  |  |  |  |
| --- | --- | --- | --- | --- | --- | --- | --- | --- | --- | --- | --- | --- | --- | --- |
|  | | \|  \| *Dependent Variable is language gender stereotype about* \| \| --- \| --- \| | | | | | | | | | | | | |
|  | | **Career-family, Math-lib. arts, Science-arts** | | **Career- family** | **Math- liberal arts** | | **Science- arts** | | **Career-family, Math-lib. arts, Science-arts** | | **Career- family** | **Math- liberal arts** | **Science- arts** |  |
|  | |  | |  |  | |  | |  | |  |  |  |  |
| **Individualism** | | 0.599*** | | 0.585*** | 0.241** | | 0.492*** | |  | |  |  |  |  |
|  | | (0.0993) | | (0.101) | (0.120) | | (0.108) | |  | |  |  |  |  |
| **GNI** | |  | |  |  | |  | | 0.558*** | | 0.569*** | 0.203* | 0.411*** |  |
|  | |  | |  |  | |  | | (0.103) | | (0.102) | (0.121) | (0.113) |  |
| Constant | | -7.45e-10 | | -3.80e-09 | 1.42e-09 | | 0 | | 2.26e-10 | | -2.82e-09 | 1.78e-09 | 7.28e-10 |  |
|  | | (0.0985) | | (0.0999) | (0.119) | | (0.107) | | (0.102) | | (0.101) | (0.121) | (0.112) |  |
|  | |  | |  |  | |  | |  | |  |  |  |  |
| Observations | | 67 | | 67 | 67 | | 67 | | 67 | | 67 | 67 | 67 |  |
| R-squared | | 0.359 | | 0.342 | 0.058 | | 0.242 | | 0.312 | | 0.323 | 0.041 | 0.169 |  |

Notes. The table presents estimates of linear regressions of country-level gender stereotypes (based on semantic similarity in Wikipedia corpora) on country levels of individualism (Hofstede's measure of individualism) and economic development (Gross National Income, GNI). Translations of the sets of target and attribute words are obtained with ChatGPT instead of Google Translate as in the main setting (see details in Appendix A). Language gender stereotypes are about career, math and science and measured as in (1) by the Word Embedding Association Test. Columns 2-4 and 6-8 consider each stereotype separately: male-career/female-family stereotypes in columns 2 and 6, male-math/female-liberal arts stereotypes in columns 3 and 7 and male-science/female-arts stereotypes in columns 4 and 8. Col. 1 and 5 consider the three stereotypes together. The sample is restricted to countries with available data for Hofstede's measure of individualism and GNI and all variables are standardized on the regression sample. Standard errors in parentheses ^***^ *p<0.01,* ^**^ *p<0.05,* ^*^ *p<0.1*

1. A. Caliskan, J. J. Bryson, A. Narayanan, Semantics derived automatically from language corpora contain human-like biases. *Science* **356**, 183–186 (2017).

2. Y. Bengio, R. Ducharme, P. Vincent, C. Jauvin, A Neural Probabilistic Language Model.

3. D. DeFranza, H. Mishra, A. Mishra, How language shapes prejudice against women: An examination across 45 world languages. *Journal of Personality and Social Psychology* **119**, 7–22 (2020).

4. N. Garg, L. Schiebinger, D. Jurafsky, J. Zou, Word Embeddings Quantify 100 Years of Gender and Ethnic Stereotypes. *Proc. Natl. Acad. Sci. U.S.A.* **115** (2018).

5. M. Lewis, G. Lupyan, Gender stereotypes are reflected in the distributional structure of 25 languages. *Nat Hum Behav* **4**, 1021–1028 (2020).

6. P. Bojanowski, E. Grave, A. Joulin, T. Mikolov, Enriching Word Vectors with Subword Information (2017) https:/doi.org/10.48550/arXiv.1607.04606 (March 25, 2023).

7. E. Grave, P. Bojanowski, P. Gupta, A. Joulin, T. Mikolov, Learning Word Vectors for 157 Languages (2018) (February 10, 2023).

8. B. Kurdi, T. C. Mann, T. E. S. Charlesworth, M. R. Banaji, The relationship between implicit intergroup attitudes and beliefs. *Proceedings of the National Academy of Sciences* **116**, 5862–5871 (2019).

9. A. G. Greenwald, D. E. McGhee, J. L. Schwartz, Measuring individual differences in implicit cognition: the implicit association test. *J Pers Soc Psychol* **74**, 1464–1480 (1998).

10. B. A. Nosek, M. R. Banaji, A. G. Greenwald, Harvesting implicit group attitudes and beliefs from a demonstration web site. *Group Dynamics: Theory, Research, and Practice* **6**, 101–115 (2002).

11. B. A. Nosek, M. R. Banaji, A. G. Greenwald, Math = male, me = female, therefore math ≠ me. *Journal of Personality and Social Psychology* **83**, 44–59 (2002).

12. T. E. S. Charlesworth, V. Yang, T. C. Mann, B. Kurdi, M. R. Banaji, Gender Stereotypes in Natural Language: Word Embeddings Show Robust Consistency Across Child and Adult Language Corpora of More Than 65 Million Words. *Psychol Sci* **32**, 218–240 (2021).

13. G. H. Hofstede, G. J. Hofstede, M. Minkov, *Cultures and organizations: software of the mind: intercultural cooperation and its importance for survival*, 3rd ed (McGraw-Hill, 2010).

14. R. J. House, P. J. Hanges, M. Javidan, P. W. Dorfman, V. Gupta, Eds., *Culture, Leadership, and Organizations: The GLOBE Study of 62 Societies*, 1st edition (SAGE Publications, Inc, 2004).

15. T. Kaiser, Nature and evoked culture: Sex differences in personality are uniquely correlated with ecological stress. *Personality and Individual Differences* **148**, 67–72 (2019).

16. C. L. Fincher, R. Thornhill, D. R. Murray, M. Schaller, Pathogen prevalence predicts human cross-cultural variability in individualism/collectivism. *Proceedings of the Royal Society B: Biological Sciences* (2008) https:/doi.org/10.1098/rspb.2008.0094 (July 28, 2023).

1. See, e.g., https://stats.wikimedia.org/wikimedia/animations/wivivi/wivivi.html and https://stats.wikimedia.org/archive/squid_reports/2018-05/SquidReportPageViewsPerLanguageBreakdown.html [↑](#footnote-ref-1)
2. See https://en.wikipedia.org/wiki/List_of_official_languages_by_country_and_territory [↑](#footnote-ref-2)
3. See, e.g., https://stats.wikimedia.org/wikimedia/animations/wivivi/wivivi.html [↑](#footnote-ref-3)
4. See https://en.wikipedia.org/wiki/List_of_official_languages_by_country_and_territory [↑](#footnote-ref-4)
